# Emergence of Scale-Free Close-Knit Friendship Structure in Online Social Networks

**DOI:** 10.1371/journal.pone.0050702

**Published:** 2012-12-14

**Authors:** Ai-Xiang Cui, Zi-Ke Zhang, Ming Tang, Pak Ming Hui, Yan Fu

**Affiliations:** 1 Web Sciences Center, University of Electronic Science and Technology of China, Chengdu, People's Republic of China; 2 Institute for Information Economy, Hangzhou Normal University, Hangzhou, People's Republic of China; 3 Department of Physics, The Chinese University of Hong Kong, Shatin, Hong Kong, People's Republic of China; Wake Forest School of Medicine, United States of America

## Abstract

Although the structural properties of online social networks have attracted much attention, the properties of the close-knit friendship structures remain an important question. Here, we mainly focus on how these mesoscale structures are affected by the local and global structural properties. Analyzing the data of four large-scale online social networks reveals several common structural properties. It is found that not only the local structures given by the indegree, outdegree, and reciprocal degree distributions follow a similar scaling behavior, the mesoscale structures represented by the distributions of close-knit friendship structures also exhibit a similar scaling law. The degree correlation is very weak over a wide range of the degrees. We propose a simple directed network model that captures the observed properties. The model incorporates two mechanisms: reciprocation and preferential attachment. Through rate equation analysis of our model, the local-scale and mesoscale structural properties are derived. In the local-scale, the same scaling behavior of indegree and outdegree distributions stems from indegree and outdegree of nodes both growing as the same function of the introduction time, and the reciprocal degree distribution also shows the same power-law due to the linear relationship between the reciprocal degree and in/outdegree of nodes. In the mesoscale, the distributions of four closed triples representing close-knit friendship structures are found to exhibit identical power-laws, a behavior attributed to the negligible degree correlations. Intriguingly, all the power-law exponents of the distributions in the local-scale and mesoscale depend only on one global parameter, the mean in/outdegree, while both the mean in/outdegree and the reciprocity together determine the ratio of the reciprocal degree of a node to its in/outdegree. Structural properties of numerical simulated networks are analyzed and compared with each of the four real networks. This work helps understand the interplay between structures on different scales in online social networks.

## Introduction

In recent years, an increasing number of online social systems (*e.g.*, *YouTube* and *Facebook*) have been attracting wide attention from different fields [1]–[3]. Online social networks provide a platform for web surfers to make acquaintance with congenial friends [4], exchange photos and personal news [5], share videos [6], establish communities or forums on focused issues [7], etc. These online interactive behaviors, which partly reflect real-life social relationships among people, provide an unprecedented opportunity to study and understand the dazzling characteristics of real-life social systems [8], [9].

Complex network theory has been proven to be a powerful framework to understand the structure and dynamics of complex systems [10]–[16]. Online social systems have been treated as undirected networks [17], [18], which have been applied successfully in exploring various systems [10]. This simplification, however cannot describe the asymmetric interactions among users. Taking *Flickr* as an example, if a user 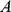 designates another user 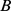 as a friend, user 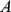 can see the photos of user 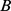, but not the other way round unless user 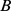 also designates user 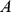 as his friend. Technically, an asymmetric interaction represents one directed link, and many online social systems are thus directed networks in nature. The directionality of links is important in characterizing the functioning of many systems, *e.g.*, leadership structure of social reputation [19], [20], reciprocal behavior in evolutionary games [21], information hierarchy of the World Wide Web [22], [23], citation relationship of scientific publications [24], [25], etc. Much effort has been devoted to understanding the structural properties of these directed networks, including the indegree and outdegree distributions [26], average shortest distance [26], degree correlation [27], and community structure [28]–[30]. Correspondingly, there are many models proposed for the underlying mechanisms of the statistical properties. Dorogovtsev *et al.* [31] generalized the Barabási-Albert(BA) model [32] and obtained the exact form of the indegree distribution of growing networks in the thermodynamic limit. Krapivsky *et al.* [33] introduced a directed network model that generates correlated indegree and outdegree distributions. Zhou *et al.* [20] argued that the “good get richer” mechanism would facilitate the emergence of scale-free leadership structure in online social networks.

Up to now, most of the work on complex networks can be classified into studies on three scales: the local scale based on the single node properties (through statistical distributions), the macro-scale based on the global properties of networks (with global parameters), and the mesoscale based on properties due to a group of nodes (via modular properties) [34]–[36]. However, a majority of studies focused on the first two scales. In view of the significant role of modularity in the functionality of real networks, it has become increasing important to study the mesoscale structures. Communities and motifs are two key mesoscale structures of real complex networks. Community structures at mesoscale level are ubiquitous in a variety of real complex systems [37], [38], such as *Facebook*, *YouTube*, and *Xiaonei*. There are more connections among members of the same community than among members in different communities. Lancichinetti *et al.* analyzed the statistical properties of communities in five categories of real complex networks, and found that communities detected in networks of the same category display similar structural characteristics [39]. Motifs, which are defined as subgraphs that occur much more often than expected in a random network, play a significant role in our understanding of the interplay between the structures and dynamics of real complex networks [40]–[45].

**Figure 1:**
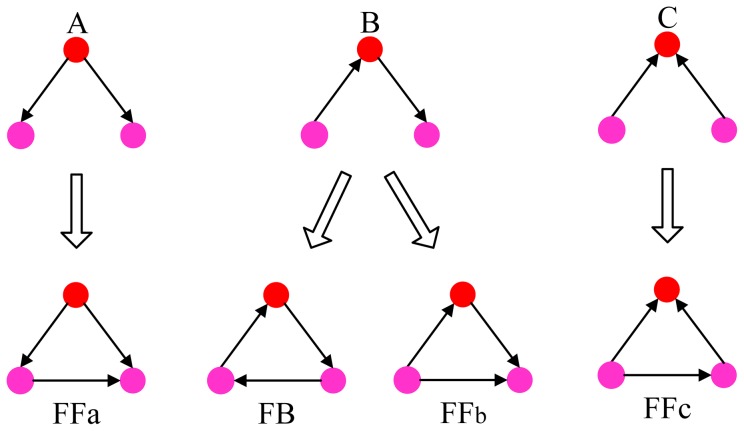
Three possible unclosed triples and four basic closed triples for a focal node (red). The basic closed triples correspond to one feedback (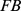) loop and three feedforward (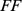) loops. The three feedforward loops differ in the indegree 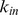 of the focal node: 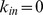 for 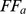, 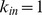 for 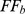 and 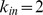 for 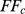. The numbers of the three feedforward loops are equal because every 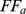 loop from the perspective of the focal node constitutes a 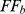 loop and a 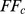 loop from the perspective of the another two nodes, but the loops may arise from different growth histories.

In spite of the structural features revealed at the three scales, understanding the interplay between the different scales has remained a major challenge [34]–[36]. In the present work, we study how the close-knit friendship structures of online social networks at the mesoscale level and the structural properties at the two other scales are affecting each other. In social networks, the close-knit friendship structure describes the closest unit, which is usually represented by the closed triples. In a directed network, there are 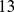 different possible three-node subgraphs [41]. For situations without reciprocal links, a focal node has three possible unclosed triples. Each unclosed triple can be closed by adding a directed link between the two unconnected nodes, giving rise to four types of closed triples as shown in Figure 1 [44], [45]. The four closed triples fall into two groups: one is a feedback (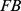) loop and the three others are feedforward (*i.e.*, 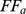, 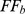, and 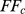) loops. Structurally, the roles of three nodes in the 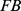 loop are equivalent, but it is not the case in the 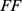 loops. Any 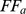 loop (from the perspective of the focal node) becomes a 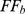 loop for another node and a 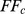 loop for the third node, and thus the numbers of three feedforward loops are equal in directed networks. Compared to the unclosed triples, the closed triples play a more important role in dynamical processes on online social networks [46], [47], such as opinion formation [48], game dynamics [49], and cooperation evolution [50].

In online social networks, the closed triples are a good indicator of close-knit friendships among people. To understand the mesoscale structural properties of online social networks, we analyze data of popular online social networks, establish the empirical facts, and introduce a directed network model. We analyze four large-scale online social networks, namely *Epinions*, *Slashdot*, *Flickr*, and *Youtube*, and establish that the distributions in each scale follow a similar power law. We propose a simple directed network model incorporating two processes: external reciprocation and internal evolution. Theoretical analysis shows that the distributions of four closed triples display almost identical scaling laws due to the negligible degree correlations, and the distribution exponents depend only on one global parameter - the mean in/outdegree. Simulation results based on the model are basically consistent with both the empirical results and theoretical analysis.

## Results

### Empirical Results

We first analyze four representative directed online social networks and establish the empirical features. As listed in Table 1, these four datasets are: (i) *Epinions* Social Network (ESN, http://snap.stanford.edu/data/soc-Epinions1.html) [51]: a who-trust-whom online social network of a general consumer review site *Epinions.com* in which members can decide whether to “trust” each other or not, and subsequently all the trusted relationships form a so-called social trust network. (ii) *Slashdot* Social Network (SSN, http://snap.stanford.edu/data/soc-Slashdot0902.html) [51]: a friendship network of a technology-related news website *Slashdot.com*. Nodes are the users and links represent the friendships among the users. (iii) *Flickr* Social Network (FSN, http://socialnetworks.mpi-sws.org/data-imc2007.html) [52]: a friendship network of a photo-sharing site *Flickr.com* that allows users to designate others as “contacts” or “friends” and track their activities in real time. This network contains all the friendship links among the users of Flickr. (iv) *YouTube* Social Netowrk (YSN, http://socialnetworks.mpi-sws.org/data-imc2007.html) [52]: a friendship network of a popular video-sharing website *YouTube.com* on which users can upload, share and view videos. The nodes in the network are the users of YouTube, and a directed link is established from a user 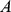 to a user 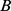 when user 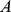 declares user 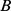 as a friend. Table 1 summarizes the basic global features of the four online social networks. These networks all show a large reciprocity 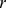, defined by 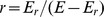 [53] with 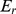 and 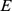 being the numbers of reciprocal links and single directed links, respectively. Note that a reciprocal link contributes two single directed links. For example, 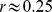 for ESN, 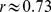 for SSN, 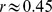 for FSN, and 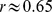 for YSN.

**Table 1:** Basic statistics of the four online social network datasets.

Data sets	Epinions	Slashdot	Flickr	YouTube
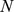	75,879	82,168	1,715,255	1,138,499
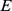	508,825	870,161	22,613,980	4,945,382
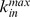	3035	2552	16255	25519
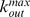	1801	2510	26185	28644
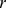	0.25	0.73	0.45	0.65
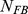	740,310	899,316	435,829,822	5,320,127
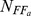	3,586,403	2,881,727	1,667,179,686	16,287,794

Properties of each network: number of users 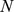, number of directed links 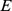, reciprocity 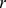, number of feedback (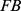) loop 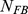, number of feedforward loops 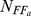. The numbers of the three feedforward loops (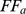, 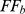, 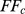) are equal, because every 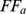 loop from the perspective of the focal node constitutes a 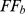 loop and a 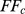 loop from the perspective of the another two nodes.

**Figure 2:**
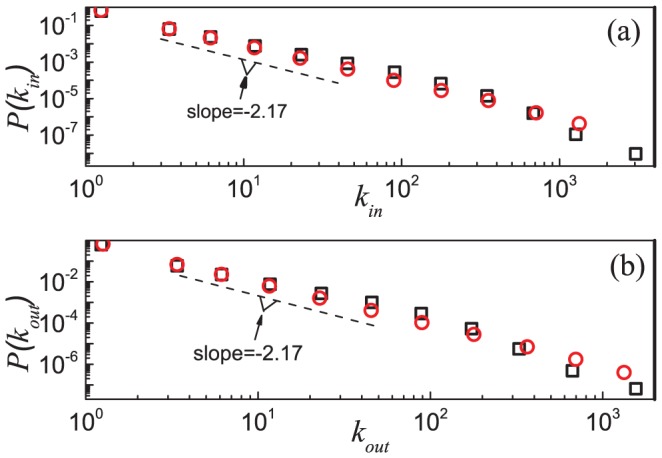
Indegree (a) and outdegree (b) distributions of the Epinions social network (black squares) and simulation results (red circles) based on the model. The dashed lines in both panels have a slope 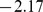 as the analytic results in Eqs. (17) and (31) suggested. The simulated network is generated by the model with the parameters 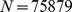, 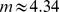 and 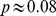, as determined by the mean degree 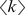 and reciprocity 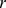 of the Epinions social network. Data points are averages over the logarithmic bins of the indegree 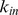 and outdegree 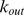, respectively.

**Figure 3 pone-0050702-g003:**
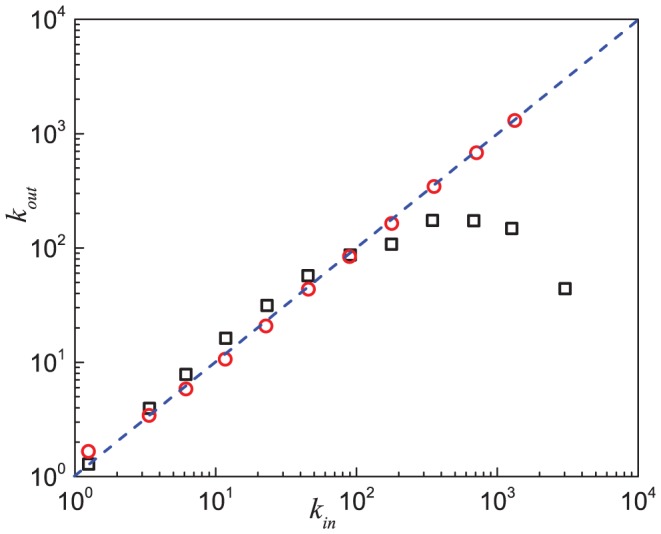
Relationship between the indegree and the outdegree of nodes in the Epinions social network and the model. Results of the Epinions social network (black squares) and simulation results (red circles) based on the model are shown. The blue dash line represents the relation function 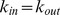. Data points are averages over the logarithmic bins of the indegree 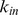.

We also studied the local-scale structural properties of these social networks via statistical distributions. The results of ESN are presented as an example. Figure 2 shows the indegree and outdegree distributions (black squares) on a log-log plot. The data span more than two decades. The distributions follow a power law with approximately the same exponent, *i.e.*, 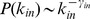 and 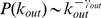, with 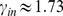 and 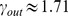 obtained by the maximum likelihood estimation [54], [55]. More details about the power-law fits are given in Table S1 of Appendix S1. Figure 3 shows that the indegree 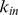 of each node is nearly proportional to its outdegree 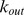 (also see Figures S4, S5, S6 of Appendix S1), which is consistent with the similar scaling law of their distributions. In growing networks, the fat-tail power-law behavior in the degree distribution suggests that directed links are not drawn toward and from existing users uniformly. Mislove *et al.* showed that there is a positive correlation between the number of links a user has and its probability of creating or receiving new links in online social networks [5]. This phenomenon is called “preferential attachment” [5], [32], [33], [56]. The behavior 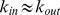 for any node implies that a node with large 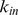 has a strong ability to attract links from other nodes and also a strong tendency to link to other nodes. This is reminiscences of the product 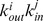 used in the prediction of a link between the nodes 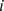 and 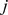 [57], *i.e.*, a larger product gives a larger probability of having a directed link from 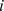 to 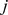. These results lead us to incorporate a preferential attachment mechanism related to 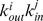 into the mechanism of how the links grow in a network.

**Figure 4 pone-0050702-g004:**
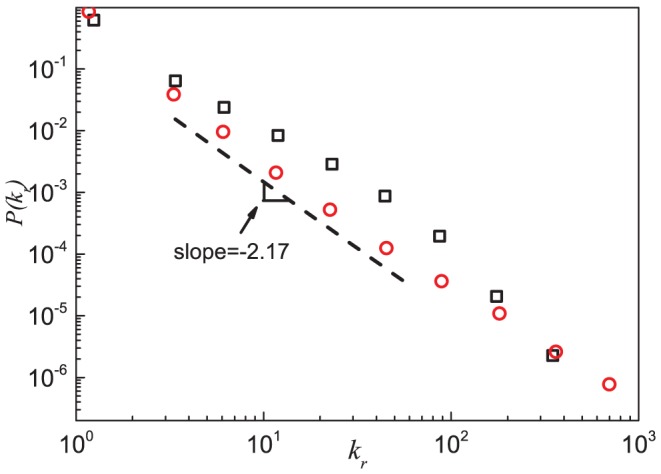
Reciprocal degree distributions of the Epinions social network and the model. Results of the Epinions social network (black squares) and simulation results (red circles) based on the model are shown. Analytic treatment (see Eqs. (17) and (31)) suggests a scaling behavior with an exponent 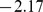, as shown by the dash line. Data points are averages over the logarithmic bins of the reciprocal degree 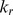.

**Figure 5 pone-0050702-g005:**
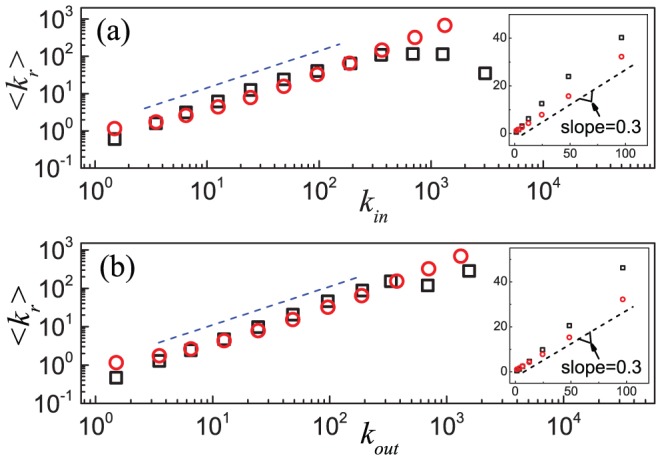
Mean reciprocal degree of nodes with (a) the same indegree and (b) the same outdegree in the Epinions social network and in the model. Results of the Epinions social network (black squares) and simulation results (red circles) based on the model are shown in a log-log scale in the main panels. Analytic treatment suggests that 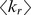 is linearly dependent on 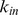 and 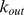, and the blue dash lines of slope 1 show its dependence. The inset in each panel shows the results in a linear scale and the dash line has a slope of 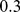, as given by Eqs. (20) and (31). Data points are averages over the logarithmic bins of the indegree 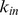 and outdegree 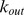, respectively.

The reciprocal degree is the number of reciprocal links that a node possesses. Figure 4 shows that the reciprocal degree distribution also follows a power law 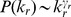 with an exponent 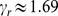 as examined by the maximum likelihood estimation [54], [55], similar to that of the indegree and outdegree distributions. Figure 5 shows that the mean reciprocal degree of the nodes with the same indegree 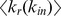 is approximately linearly proportional to the indegree 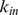 (also see Figures S10, S11, S12 of Appendix S1), *i.e.*, 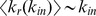, and in a similar fashion 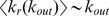, implying that the probability that a randomly chosen directed link happens to be a reciprocal link is roughly a constant. All these features are consistent with the observation that the indegree, outdegree, and reciprocal degree distributions all follow a similar exponent.

**Figure 6 pone-0050702-g006:**
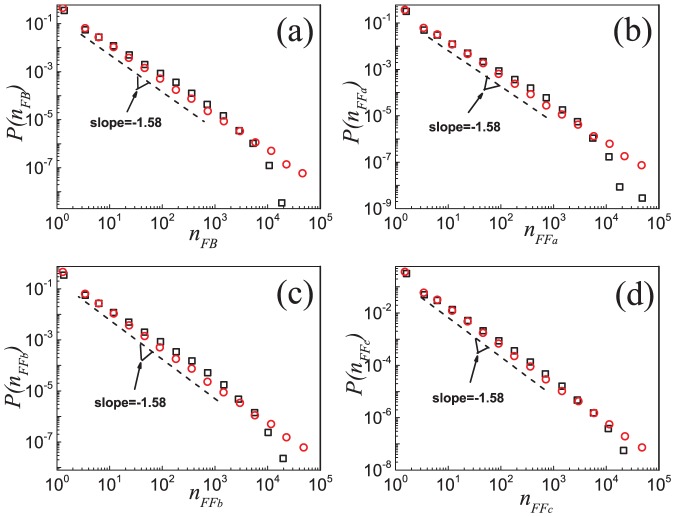
Distributions of four basic closed triples in the Epinions social network and the model. Distributions of closed triples corresponding to (a) 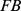, (b) 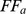, (c) 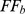, and (d) 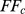 loops in the Epinions social network (black squares) and in the simulated network based on the model (red circles). Analytic treatment (see Eqs. (30) and (31)) suggests a scaling behavior with an exponent 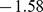, as shown by the dash lines. Data points are averages over the logarithmic bins of the 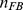, 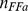, 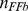 and 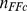, respectively.

For mesoscale structures, we focus on the four closed triples *i.e.*, 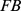, 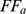, 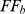 and 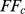. As the numbers of three feedforward loops are equal, *i.e.*, 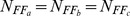, we only look at the total numbers of 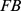 and 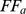 closed triples. For ESN, 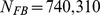 and 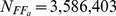 as shown in Table 1. Considering the feedforward loops as the same up to the permutation of the focal node, it is interesting to see that 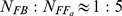. This implies the existence of some underlying mechanism. Since the indegree and outdegree distributions are heterogeneous, we study the numbers of the four closed triples (*i.e.*, 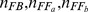, and 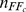) at different nodes and their distributions. Figure 6 shows that, although the numbers of feedback and feedforward loops are different, their distributions follow similar scaling laws, *i.e.*, 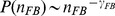 and 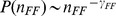, with 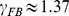, 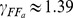, 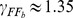 and 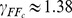 as determined by the maximum likelihood estimation [54], [55]. More details on the exponents are given in Table S1 of Appendix S1. Moreover, although the numbers of three feedforward loops are equal, their distributions look slightly different in detail. This is a phenomenon worthy of further research.

**Figure 7 pone-0050702-g007:**
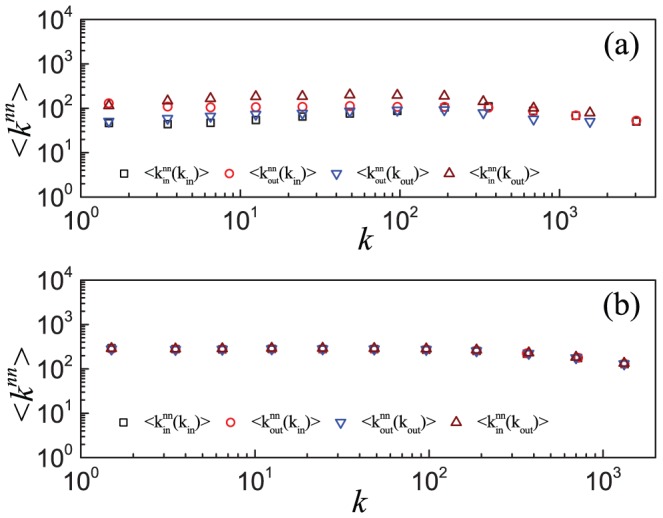
Degree correlations in the Epinions social network and the model. Results of degree correlations as measured by four quantities corresponding to the average nearest neighbor degree 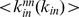 (squares), 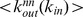 (circles), 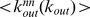 (triangles), and 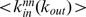 (inverted triangles) for (a) the Epinions social network and (b) simulated network based on the model. Data points are averages over the logarithmic bins of the indegree 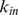 or outdegree 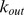.

**Figure 8 pone-0050702-g008:**
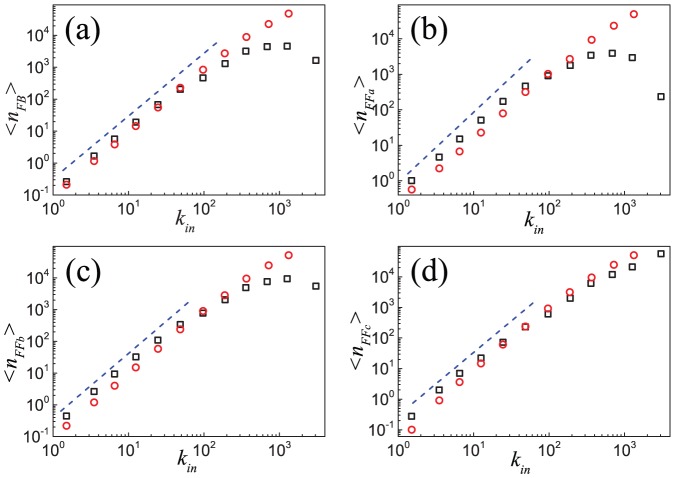
Mean number of the four closed triples for nodes with the same indegree in the Epinions social network and the model. Results for the mean number of closed triples corresponding to (a) 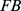, (b) 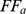, (c) 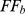, and (d) 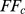 loops for nodes with the same indegree are shown for the Epinions social network (black squares) and simulated network (red circles) based on the model. Analytic treatment (see Eq. (28)) gives a scaling behavior with an exponent 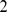, as indicated by the dash lines. Data points are averages over the logarithmic bins of the indegree 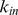.

**Figure 9:**
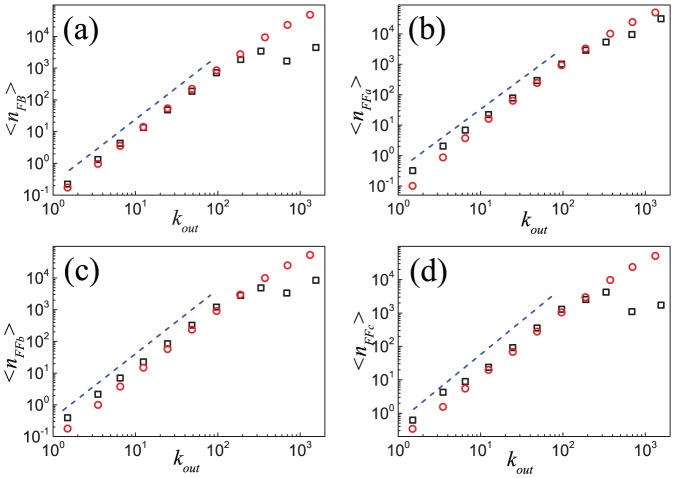
Mean number of the four closed triples for nodes with the same outdegree in the Epinions social network and the model. Results for the mean number of closed triples corresponding to (a) 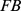, (b) 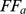, (c) 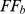, and (d) 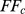 loops for nodes with the same outdegree are shown for the Epinions social network (black squares) and simulated network (red circles) based on the model. Analytic treatment (see Eq. (28)) gives a scaling behavior with an exponent 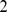, as indicated by the dash lines. Data points are averages over the logarithmic bins of the outdegree 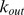.

To understand this phenomenon, we consider the three unclosed triples in Figure 1. For a node with indegree 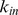 and outdegree 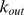, there are 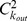 unclosed triples 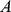, 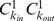 unclosed triples 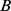, and 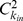 unclosed triples 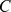 when reciprocal links are forbidden, where 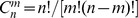 denotes the binomial coefficient. These unclosed triples would generate closed triples in the ratio 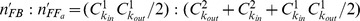. Accounting for all the nodes, we can obtain the total number of optional closed triples 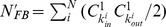 and 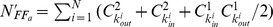, respectively. Assuming there is no degree correlation and making use of 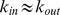, we have 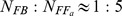, which is basically consistent with the ratio found in ESN. The assumption of no degree distribution is supported by the results in Figure 7(a), in which the network shows a very weak degree correlation over two decades that can be treated almost as no degree correlation (further quantitative evidence is given by the Pearson correlation coefficient in Table S2 of Appendix S1) [58]. In this case, the number of closed triples at a node depends only on its indegree 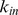 and outdegree 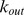, *i.e.*, 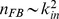 and 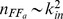 for large 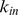, 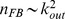 and 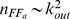 for large 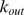. This behavior is confirmed in Figure 8 and Figure 9 (also see Figures S19, S20, S21, S22, S23, S24 of Appendix S1). This also gives the reason why the distributions of four closed triples follow similar scaling laws. Results of analyzing the other three networks (*i.e.*, *Slashdot*, *Flickr* and *YouTube*) also exhibit similar phenomena (see Figures S1, S2, S3, S4, S5, S6, S7, S8, S9, S10, S11, S12, S13, S14, S15, S16, S17, S18, S19, S20, S21, S22, S23, S24 of Appendix S1).

### Directed Network Model

We propose a growing network model with node and link creation processes incorporating link directionality that reproduces the empirical features. In the model, we consider two evolutionary ingredients: reciprocation and preferential attachment. On one hand, many empirical results show that the reciprocity 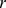 of online social networks is much greater than in sparse random directed networks with 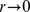 [5], [53]. Our results of 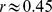 of FSN and 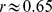 of YSN provide further evidence. The high reciprocity implies that there is a good chance that the creation of a directed link prompts the establishment of a reversed link. For example, users of Flickr often respond to an incoming link by quickly establishing a reversed link as a matter of courtesy [5]. Thus, reciprocation is believed to be an independent growth mechanism in large-scale online social networks. On the other hand, preferential attachment has been proven to be an important and basic growing mechanism in online social networks [5], [32], [33], [56]. Users with large indegrees and outdegrees are more likely to receive incoming links and create outgoing links, respectively. This motivated us to incorporate a preferential attachment mechanism depending on the product 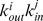 in creating new links.

The model starts with an initial seed consisting of 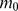 nodes. At each time step, a new node is added and 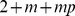 new directed links are introduced according to two processes: external reciprocation and internal evolution.

1. External reciprocation. The new node in every time step establishes a new directed link with an existing nodes 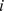 in the network with a probability
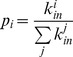
(1)proportional to the indegree 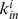 of node 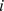. To incorporate the reciprocation mechanism, the node 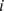 that receives the link creates a reversed link to the new node. Consequently, a reciprocal link is created between these two nodes. This mechanism is reasonable in that a strong motivation of a new user joining a social network is to get connected to and interact with someone already in the network. As we shall see, this process can be treated conveniently in the mathematical analysis of the model.
2. Internal evolution. In each time step, 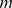 new directed links, representing the activity of the network, are created among the existing nodes according to the preferential attachment mechanism. Consider two unconnected nodes 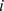 and 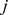 up to that time step, a new directed link from node 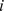 to node 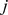 is created with the probability
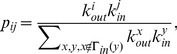
(2)where 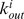 and 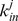 are the outdegree of node 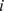 and the indegree of the target node 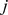, respectively, and 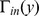 in the normalization factor is the set of incoming neighbors of node 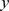 at that time step. This attachment probability is proportional to the product 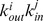. The larger the product is, the greater probability a new directed link is created between them. For each of the new directed links created, a reversed link will be established with the reciprocation probability 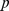. Therefore, 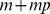 directed links are introduced into the network through internal evolution in each time step. It should be noted that multiple links between two nodes and self-connections are prohibited in the model.

## Materials and Methods

### Rate Equation Analysis

We first analyze the indegree and outdegree distributions of the model. After 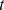 steps, the growing directed network has 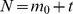 nodes and 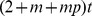 directed links, where the tiny number of initial links in the seed are ignored. Meanwhile, the sum of indegree and the sum of outdegree are equal, *i.e.*, 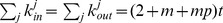. For a sparse network with mean indegree 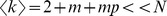, we have 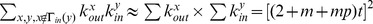 so that Eq. (2) can be approximated by
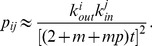
(3)Consider the creation of one new directed link via the internal evolution at step 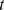. The probability 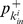 that the indegree 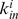 of node 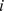 increases by one due to the creation of one link is
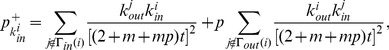
(4)where the first term gives the probability that the node 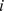 receives a new incoming link from one of the other nodes and the second term gives the probability that a reversed link is created back to node 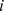 when a new directed link was created from node 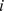 to some node 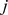. According to 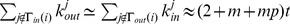, 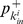 is approximately given by
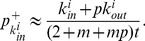
(5)Similarly, the probability 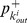 that the outdegree 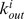 of node 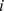 increases by one due to the creation of one link is
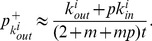
(6)Equations for the rate of change of the expected indegree 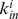 and outdegree 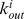 can then be written down. Taking 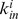 and 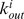 as continuous variables, the dynamical equations are
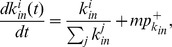


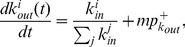
(7)where the first term in the equations comes from the newly added node in a time step. The difference of the two equations gives
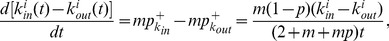
(8)where Eqs. (5) and (6) have been used. Let 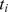 be the time that the node 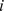 is introduced, *i. e.*, 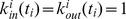. It follows from Eq. (8) that 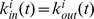 at any time 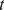. Although the expected value of the difference between indegrees and outdegrees of a node does not grow over time mathematically, the difference does exist in a particular realization of the model in simulations. Eq. (7) and the initial condition 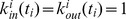 gives
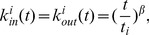
(9)where 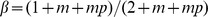. The indegree and outdegree of the nodes both grow over time in the same functional form, with older nodes having higher indegrees and outdegrees.

Let 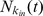 and 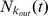 be the number of nodes with expected indegree 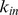 and outdegree 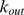 at the time step 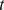, respectively. The rate equation of 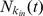 is then given by
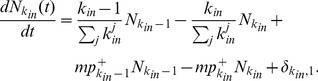
(10)The first and third terms on the right-hand side account for the increase of 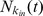 due to the external reciprocation and internal evolution, respectively; and the second and fourth terms account for the decrease due to the processes. The last term accounts for the introduction of a new node with indegree 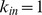 at time 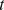. Eq. (10) is valid for all 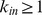.

After many steps 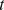, there are 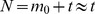 nodes in the network. In the asymptotic limit, we substitute 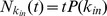, where 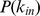 is the indegree distribution [59], and 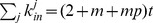 into Eq. (10) to obtain the simple recursive relation
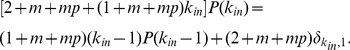
(11)Using the initial condition that 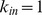 at the time that a node was introduced, the solution of Eq.(11) is
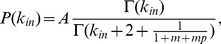
(12)where 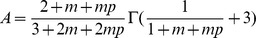 and 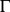 is the Euler gamma function. Using the asymptotic form 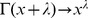 as 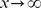, we can extract the scaling form
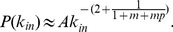
(13)


Similarly, the rate equation of 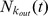 is given by
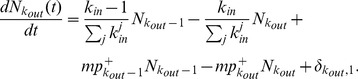
(14)The first (second) and third (fourth) terms on the right-hand side account for the increase (decrease) in 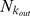 due to the external reciprocation and internal evolution, respectively; and the last term accounts for the introduction of a new node with 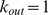 at time 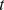. Substituting 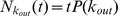, where 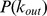 is the outdegree distribution, and 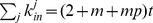 into Eq. (14), the recursive relation for 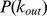 is
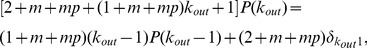
(15)which is identical to Eq. (11) for 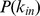. It follows that
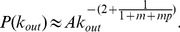
(16)The results show that the expected indegree and outdegree grow over time following the same functional form of Eq. (9), and the indegree and outdegree distributions follow the same scaling law with an exponent
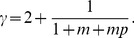
(17)


Next, we consider the reciprocal degree distribution 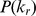. For a node 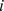 with 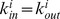, 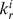 satisfies the dynamical equation
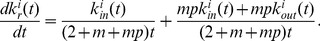
(18)Substituting Eq. (9) into Eq. (18) and using the initial condition that 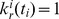 at the time that node 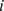 was introduced, the solution to Eq. (18) is
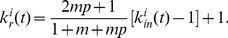
(19)For large 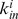, we have
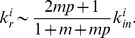
(20)Using 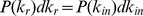, the distribution 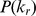 follows
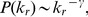
(21)where 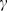 is given by Eq. (17) as for the indegree and outdegree distributions.

Furthermore, we analyze the degree correlations between connected nodes by the rate equation approach. Let 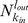 be the number of links that originate from a node with an expected outdegree 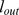 to a node with an expected indegree 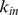 [60]. Generally, 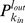 is defined for 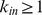 and 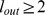. The quantity 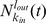 evolves according to
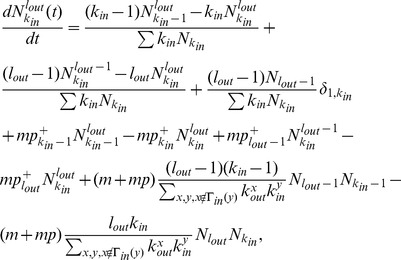
(22)


where the first two terms on the right-hand side account for the changes due to the introduction of a new node, including the gains when the new node is connected to a node with indegree 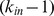 (outdegree 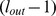) which is already connected to a node with outdegree 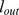 (indegree 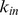), and the losses when the new node is connected to either end of a link that connects a node with outdegree 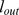 and another node with indegree 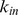. The third term accounts for the gain in 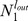 due to the addition of the new node. The remaining terms take into account the changes due to the internal evolution process with the introduction of 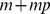 directed links.

Asymptotically, 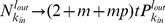, 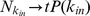 and 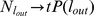. Considering 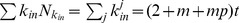 and 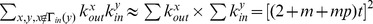, Eq. (22) gives a recursive relation
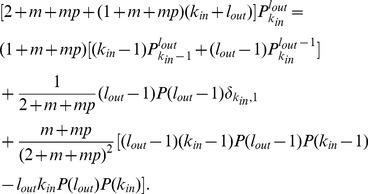
(23)Solving Eq. (23) directly for 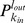 is difficult, however, it is observed that decomposing 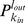 into
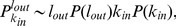
(24)with 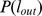 given by Eq. (16) and 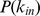 given by Eq. (13) satisfies Eq. (23) in the scaling regime, as one can readily show by substituting Eq. (24) into Eq. (23) and taking the limits of 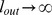 and 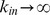. Eq. (24) implies that there is no degree correlation, a feature that is supported by the empirical results in Figure 7 for ESN over a wide range of degrees (also see Figures S16, S17, S18 of Appendix S1). It also follows from 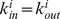 and Eq. (24) that 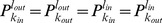. Interpreting 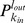 as a joint probability, the lack of degree correlation as expressed in Eq. (24) implies that the conditional probability
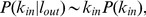
(25)which is independent of 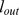. For a node 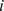 with large 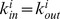, the average nearest neighbor function can be calculated as
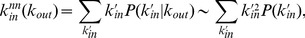
(26)which is also independent of 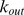. This is consistent with the behavior of 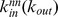 in ESN, as shown in Figure 7.

The number of 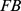 loops can be formally written as [61]

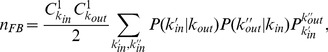
(27)where 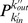 is the probability that a link connects a node with outdegree 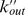 to a node with indegree 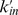. The lack of degree correlations makes the summations independent of 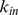 and 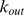, and thus 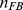 scales as
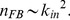
(28)Similarly, the numbers of four closed triples 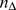 at a node with large indegree and outdegree follow the scaling behavior 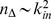 or 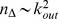. Combining 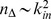 with Eq. (13) (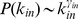), the distributions of four closed triples have the same scaling behavior as follows:
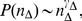
(29)where the exponent 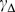 can be readily found by using 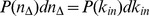 to be
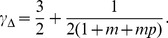
(30)The exponent 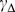 is determined by the parameters 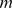 and 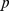 and it falls into the range 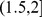.

### Simulation Results

We also carried out numerical simulations to study the structural properties of the model and compared results with data of real online social networks. The activity 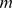 and reciprocation probability 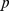 are two important parameters of the model. They determine the reciprocity 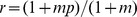 and mean indegree 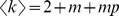 of simulated networks. In order to compare results with real online social networks, we take three parameters from real data, namely the number of nodes 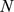, the reciprocity 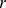 and the mean indegree (outdegree) 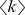, and determine the parameter 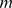 and 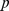 in the model through
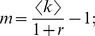


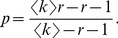
(31)


Taking ESN as an example, we have 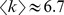, 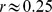, and 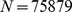. The model parameters are then fixed at 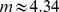 and 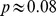 according to Eq.(31). With the values of 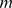 and 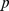, a network of 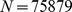 nodes is simulated. For a non-integer value of 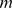, it is implemented in a probabilistic way. For ESN with 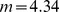, for example, the initiation of the fifth new directed link through the internal evolution process is implemented with a probability 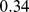 after establishing four new directed links in every time step. The structural properties of the simulated network are analyzed for each of the quantities studied for the real data. Results are shown in Figures 2–9 as red circles for comparison (also see Figures S1, S2, S3, S4, S5, S6, S7, S8, S9, S10, S11, S12, S13, S14, S15, S16, S17, S18, S19, S20, S21, S22, S23, S24 of Appendix S1). The model basically reproduces the key properties of ESN.

For the indegree and outdegree distributions (see Figure 2) and the reciprocal degree distribution (see Figure 4), the simulation results also show similar scaling law, with the exponents 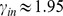, 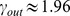 and 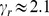 determined by the maximum likelihood estimation [54], [55] (see Table S1 of Appendix S1 for more detail). These values are slightly larger than the corresponding values of the exponents in ESN. According to Eqs. (17), (21) and (31), these exponents are equal and the theoretical value is 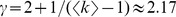. Note that the rate equation analysis assumes an infinite system. The difference between the simulated results and the theoretical value comes from the finite size of simulated network, as well as the approximations made in getting at the values of the exponent. The indegree and outdegree distributions of simulated network are in reasonable agreement with the empirical results of ESN. The model, however, gives a reciprocal degree distribution smaller than the ESN empirical results over a wide range of 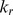. This discrepancy implies that there are some network growing mechanisms in ESN that are not included in the model, *e.g.*, different reciprocation probabilities for different nodes [62]. This, together with a possibly very weak degree correlation in Figure 7 that we ignored, may be the reason for the simulation results in Figures 3 and 5 to be bigger than the empirical values for large in/outdegrees, and for the small differences in the tails in Figures 2 and 4 [63], [64].

**Figure 10:**
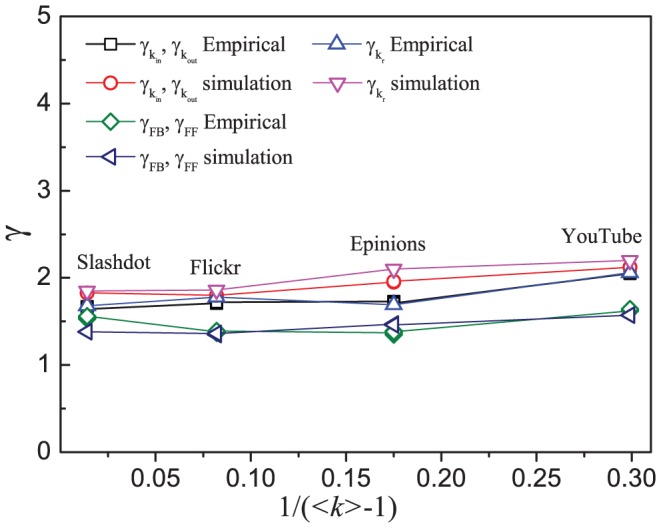
Values of the 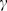
 **-exponents for various distributions.** Values of the 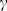-exponents for the various distributions as determined by the maximum likelihood estimation against 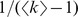 for each of the four large-scale online social networks and the corresponding simulated networks based on the model. The lines are only guides to the eye.

For the distributions of the four closed triples, the distributions from simulations follow a power-law behavior with almost the same exponent (see Figure 6), where 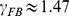, 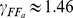, 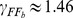 and 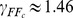 as determined by the maximum likelihood estimation. These values are slightly larger than the exponents found in ESN. Theoretically, 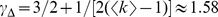 according to Eqs. (30) and (31). We note that the theoretical values of both 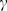 and 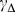 depend only on the mean indegree 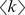, which in turn is determined by the two model parameters 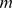 and 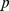. Figure 10 shows the values of all the 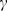-exponents of the distributions for the four online social networks and the corresponding simulated networks, which are determined by the maximum likelihood estimation.

The two parameters 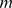 and 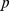 affect the reciprocal degree of nodes 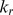 through Eq.(20). Substituting Eq. (31) into Eq. (20), we have 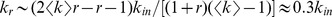 for ESN. The reciprocal degree 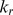 of a node and its 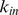 are related by a factor depending on the two global parameters 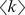 and 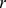. This linear relationship between 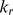 and 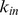 (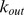) with a slope 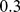 is observed in simulation results, as shown in Figure 5, but the ESN data show a faster increase of 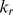 with 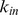 and 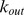. When the network has a larger reciprocity, such as 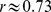 for *Slashdot*, 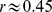 for *Flicker*, and 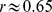 for *YouTube*, a better agreement is observed (see Figures S10, S11, S12 of Appendix S1). Despite some small differences in the tail in Figures 8 and 9, which may be caused by local proximity bias in link creation [5], simulation results for the dependence of the number of closed triples with 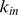 and 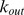 are basically in accordance with empirical results.

More comparison of results between the model and large-scale online social networks are given in Appendix S1 (see Figures S1, S2, S3, S4, S5, S6, S7, S8, S9, S10, S11, S12, S13, S14, S15, S16, S17, S18, S19, S20, S21, S22, S23, S24). The results further support the notions that the two mechanisms incorporated in our model provide a potential explanation of the local and mesoscale structures in these online social networks.

## Discussion

With the advancement in information technology, online social systems become an increasingly important part of modern life. It is, therefore, of great significance to study the structures and dynamics of these systems. In this study, we focused on the local scale, mesoscale and macroscale structural properties of online social networks, especially the influence of properties on the local scale and macroscale on the mesoscale structures. We analyzed the data and extracted the local scale and macroscale structural properties of four large-scale online social networks. It was found that the indegree and outdegree distributions follow a similar scaling law, which follows from the fact that 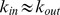 for most of the nodes. It implies that there is a preferential attachment mechanism in which the product 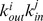 is important in the establishment of links during the evolution of online social networks. In addition, the very large reciprocity 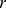 observed in these networks suggests the existence of a reciprocation mechanism in online social networks. The reciprocal degree distribution also shows a similar exponent as that of the indegree distribution due to the roughly linear relationship between the reciprocal degree 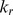 and the indegree 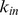 of nodes (*i.e.*, 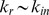), which in turn implies a fixed probability of reciprocal links between connected nodes. In the mesoscale, the close-knit friendship structures are determined by both local scale (*i.e.*, indegree and outdegree 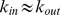) and macroscale (*i.e.*, mean in/outdegree 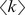) structural properties. For a node with large 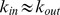, the numbers of the four closed triples show the same scaling behavior: 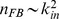 and 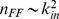, as a result of the negligible degree correlations in these networks. For all nodes, the distributions of these closed triples also follow a similar scaling law. Despite the numbers of the three feedforward loops are equal, their distributions look somewhat different in detail.

To reproduce the empirical features, we proposed and studied a simple directed network model incorporating an external reciprocation process and an internal evolution process. The two parameters in the model are the activity 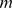 and the reciprocation probability 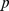. They can be inferred from the reciprocity 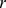 and mean indegree 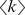 of real online social networks according to Eq.(31), so as to ensure that the simulated network and the real network have the same reciprocity and mean indegree. Analytically, we derived the structural properties in the local-scale and mesoscale. The results show that the exponents characterizing the distributions of indegree, outdegree, reciprocal degree and four closed triples depend only on the mean indegree 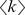, *i.e.*, 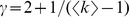 and 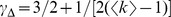. In addition, the mean indegree 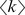 and the reciprocity 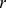 together determine the ratio of the reciprocal degree to the directed in/outdegree, *i.e.*, 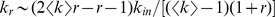. The expected indegree and outdegree of nodes in the model grow as the same function of the time that the nodes are introduced, with very old nodes having very high indegrees and outdegrees. This phenomenon, coupled with an essentially fixed rate of reciprocation, reproduces almost all the properties of the online social networks studied here.

The mesoscale structural properties reported in our work help us understand the interplay between structural properties on different scales in online social networks. More specifically, the mesoscale structures in these online social networks are determined by global parameters as well as by local distributions. This provides a useful perspective of future studies in social network analysis. Our work also provides a better understanding of the evolution of online social networks, especially the emergence of close-knit friendship structures with a scaling behavior in their distributions. The two processes (reciprocation and preferential attachment) provide a possible explanation of the mechanisms underlying the local scale and mesoscale structural properties of online social networks. The former reflects that users often respond to a new incoming link by quickly establishing a reversed link. The latter means that a well-known user with a large 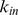 is more likely to attract new connections and an active user with a large 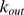 is more likely to create new connections. Our model may also be applied to other growing directed networks in which the indegree and outdgree distributions show a similar scaling behavior and the reciprocation mechanism is valid. However, the model is not applicable to the symmetric online social networks that lack the power-law degree distributions [1]–[3] (*e.g.*, Facebook), and to the WWW [33] and Wikipedia [56] as the indegree and outdegree distributions in these systems carry different exponents and the reciprocation mechanism is absent. Similarly, it does not apply to the citation network as a paper can only cite published papers, but not vice versa.

Although simulated results of our model basically reproduced the structural properties of the online social networks at different scales, the differences in the exponents characterizing the distributions and in the tails of the distributions in real online social networks (*e.g.*, Figures 4, 5, 8, 9) imply that there exist other factors, such as individual users of different reciprocation probabilities and local proximity bias, that are ignored in the model. These factors are good ingredients for future work. It is also important to study the emergence of communities in online social networks. The present work also forms the basis for the understanding of the impact of mesoscale structural properties on dynamical processes on online social networks, such as information diffusion, opinions formation, and cooperation evolution. An interesting problem for future work is to investigate whether the model can be applied to offline real social networks. Such a work would help reveal the difference between online and offline social networks.

## Supporting Information

Appendix SI Appendix to the manuscript.
(PDF)
**Table S1**
**The exponents of various distributions obtained by power-law fits of real online social networks and the simulated network based on the model using the maximum likelihood estimation.** *x_min_* is the lower bound of the range for fitting a power-law distribution, is the corresponding exponent and *KS* is the goodness-of-fit value based on the Kolmogorov-Smirnov statistic.
(PDF)
**Table S2**
**Pearson correlation coefficient.** *r*(*in; in*) quantifies the tendency of nodes with a high indegree to be connected to another node with a high indegree. The other quantities carry a similar interpretation.
(PDF)
**Figure S1**
**Indegree (a) and outdegree (b) distributions of the Slashdot social network (black squares) and simulation results (red circles) based on the model.** The dashed lines in both panels have a slope *−*2.1 as the analytic results in Eqs. (17) and (31) suggested. The simulated network is generated by the model with the parameters *N* =  82168, *m ≈*5.14 and *p ≈*0.67 as determined by the mean degree *〈k〉* and reciprocity of the Slashdot social network. Data points are averages over the logarithmic bins of the indegree *k_in_* and outdegree *k_out_*, respectively.
(PDF)
**Figure S2**
**Indegree (a) and outdegree (b) distributions of the Flickr social network (black squares) and simulation results (red circles) based on the model.** The dashed lines in both panels have a slope *−*2.08 as the analytic results in Eqs. (17) and (31) suggested. The simulated network is generated by the model with the parameters *N* =  100000, *m ≈*8.07 and *p ≈*0.39 as determined by the mean degree *〈k〉* and reciprocity of the Flickr social network. Data points are averages over the logarithmic bins of the indegree *k_in_* and outdegree *k_out_*, respectively.
(PDF)
**Figure S3**
**Indegree (a) and outdegree (b) distributions of the YouTube social network (black squares) and simulation results (red circles) based on the model.** The dashed lines in both panels have a slope *−*2.3 as the analytic results in Eqs. (17) and (31) suggested. The simulated network is generated by the model with the parameters *N* =  100000, *m ≈*4.34 and *p ≈*0.08 as determined by the mean degree *〈k〉* and reciprocity of the YouTube social network. Data points are averages over the logarithmic bins of the indegree *k_in_* and outdegree *k_out_*, respectively.
(PDF)
**Figure S4**
**Relationship between the indegree and the outdegree of nodes in the Slashdot social network and the model.** Results of the Slashdot social network (black squares) and simulation results (red circles) based on the model are shown. The blue dash line represents the relation function *k_in_* = *k_out_*. Data points are averages over the logarithmic bins of the indegree *k_in_*.
(PDF)
**Figure S5**
**Relationship between the indegree and the outdegree of nodes in the Flickr social network and the model.** Results of the Flickr social network (black squares) and simulation results (red circles) based on the model are shown. The blue dash line represents the relation function *k_in_* = *k_out_*. Data points are averages over the logarithmic bins of the indegree *k_in_*.
(PDF)
**Figure S6**
**Relationship between the indegree and the outdegree of nodes in the YouTube social network and the model.** Results of the YouTube social network (black squares) and simulation results (red circles) based on the model are shown. The blue dash line represents the relation function *k_in_* = *k_out_*. Data points are averages over the logarithmic bins of the indegree *k_in_*.
(PDF)
**Figure S7**
**Reciprocal degree distributions of the Slashdot social network and the model.** Results of the Slashdot social network (black squares) and simulation results (red circles) based on the model are shown. Analytic treatment (see Eqs. (17) and (31)) suggests a scaling behavior with an exponent *−*2.1, as shown by the dash line. Data points are averages over the logarithmic bins of the 13 reciprocal degree *k_r_*.
(PDF)
**Figure S8**
**Reciprocal degree distributions of the Flickr social network and the model.** Results of the Flickr social network (black squares) and simulation results (red circles) based on the model are shown. Analytic treatment (see Eqs. (17) and (31)) suggests a scaling behavior with an exponent *−*2.08, as shown by the dash line. Data points are averages over the logarithmic bins of the reciprocal degree *k_r_*.
(PDF)
**Figure S9**
**Reciprocal degree distributions of the YouTube social network and the model.** Results of the YouTube social network (black squares) and simulation results (red circles) based on the model are shown. Analytic treatment (see Eqs. (17) and (31)) suggests a scaling behavior with an exponent *−*2.3, as shown by the dash line. Data points are averages over the logarithmic bins of the reciprocal degree *k_r_*.
(PDF)
**Figure S10**
**Mean reciprocal degree of nodes with (a) the same indegree and (b) the same outdegree in the Slashdot social network and in the model.** Results of the Slashdot social network (black squares) and simulation results (red circles) based on the model are shown in a log-log scale in the main panels. Analytic treatment suggests that *〈k_r_〉* is linearly dependent on *k_in_* and *k_out_*, and the blue dash lines of slope 1 show its dependence. The inset in each panel shows the results in a linear scale and the dash line has a slope of 0.82, as given by Eqs. (20) and (31). Data points are averages over the logarithmic bins of the indegree *k_in_* and outdegree *k_out_*, respectively.
(PDF)
**Figure S11**
**Mean reciprocal degree of nodes with (a) the same indegree and (b) the same outdegree in the Flickr social network and in the model.** Results of the Flickr social network (black squares) and simulation results (red circles) based on the model are shown in a log-log scale in the main panels. Analytic treatment suggests that *〈k_r_〉* is linearly dependent on *k_in_* and *k_out_*, and the blue dash lines of slope 1 show its dependence. The inset in each panel shows the results in a linear scale and the dash line has a slope of 0.59, as given by Eqs. (20) and (31). Data points are averages over the logarithmic bins of the indegree *k_in_* and outdegree *k_out_*, respectively.
(PDF)
**Figure S12**
**Mean reciprocal degree of nodes with (a) the same indegree and (b) the same outdegree in the YouTube social network and in the model.** Results of the YouTube social network (black squares) and simulation results (red circles) based on the model are shown in a log-log scale in the main panels. Analytic treatment suggests that *〈k_r_〉* is linearly dependent on *k_in_* and *k_out_*, and the blue dash lines of slope 1 show its dependence. The inset in each panel shows the results in a linear scale and the dash line has a slope of 0.73, as given by Eqs. (20) and (31). Data points are averages over the logarithmic bins of the indegree *k_in_* and outdegree *k_out_*, respectively.
(PDF)
**Figure S13**
**Distributions of four basic closed triples in the slashdot social network and the model.** Distributions of closed triples corresponding to (a) *FB*, (b) *FF_a_*, (c) *FF_b_*, and (d) *FF_c_* loops in the Slashdot social network (black squares) and in the simulated network based on the model (red circles). Analytic treatment (see Eqs. (30) and (31)) suggests a scaling behavior with an exponent *−*1.55, as shown by the dash lines. Data points are averages over the logarithmic bins of the *n_FB_*, *n_FF___a__*, *n_FF___b__* and *n_FF___c__*, respectively.
(PDF)
**Figure S14**
**Distributions of four basic closed triples in the Flickr social network and the model.** Distributions of closed triples corresponding to (a) *FB*, (b) *FF_a_*, (c) *FF_b_*, and (d) *FF_c_* loops in the Flickr social network (black squares) and in the simulated network based on the model (red circles). Analytic treatment (see Eqs. (30) and (31)) suggests a scaling behavior with an exponent *−*1.54, as shown by the dash lines. Data points are averages over the logarithmic bins of the *n_FB_*, *n_FF_a__*, *n_FF_b__* and *n_FF_c__*, respectively.
(PDF)
**Figure S15**
**Distributions of four basic closed triples in the YouTube social network and the model.** Distributions of closed triples corresponding to (a) *FB*, (b) *FF_a_*, (c) *FF_b_*, and (d) *FF_c_* loops in the YouTube social network (black squares) and in the simulated network based on the model (red circles). Analytic treatment (see Eqs. (30) and (31)) suggests a scaling behavior with an exponent *−*1.65, as shown by the dash lines. Data points are averages over the logarithmic bins of the *n_FB_*, *n_FF_a__*, *n_FF_b__* and *n_FF_c__*, respectively.
(PDF)
**Figure S16**
**Degree correlations in the Slashdot social network and the model.** Results of degree correlations as measured by four quantities corresponding to the average nearest neighbor degree *< k^nn^_in_* (*k_in_*) *>* (squares), *< k^nn^_out_*(*k_in_*) *>* (circles), *< k^nn^_out_*(*k_out_*) *>* (triangles), and *< k^nn^_in_* (*k_out_*) *>* (inverted triangles) for (a) Slashdot social network and (b) simulated network based on the model. Data points are averages over the logarithmic bins of the indegree *k_in_* or outdegree *k_out_*.
(PDF)
**Figure S17**
**Degree correlations in the Flickr social network and the model.** Results of degree correlations as measured by four quantities corresponding to the average nearest neighbor degree *< k^nn^_in_* (*k_in_*) *>* (squares), *< k^nn^_out_*(*k_in_*) *>* (circles), *< k^nn^_out_*(*k_out_*) *>* (triangles), and *< k^nn^_in_* (*k_out_*) *>* (inverted triangles) for (a) Flickr social network and (b) simulated network based on the model. Data points are averages over the logarithmic bins of the indegree *k_in_* or outdegree *k_out_*.
(PDF)
**Figure S18**
**Degree correlations in the YouTube social netowrk and the model.** Results of degree correlations as measured by four quantities corresponding to the average nearest neighbor degree *< k^nn^_in_* (*k_in_*) *>* (squares), *< k^nn^_out_*(*k_in_*) *>* (circles), *< k^nn^_out_*(*k_out_*) *>* (triangles), and *< k^nn^_in_* (*k_out_*) *>* (inverted triangles) for (a) YouTube social network and (b) simulated network based on the model. Data points are averages over the logarithmic bins of the indegree *k_in_* or outdegree *k_out_*.
(PDF)
**Figure S19**
**Mean number of the four closed triples for nodes with the same indegree in the Slashdot social network and the model.** Results for the mean number of closed triples corresponding to (a) *FB*, (b) *FF_a_*, (c) *FF_b_*, and (d) *FF_c_* loops for nodes with the same indegree are shown for the Slashdot social network (black squares) and simulated network (red circles) based on the model. Analytic treatment (see Eq. (28)) gives a scaling behavior with an exponent 2, as indicated by the dash line. Data points are averages over the logarithmic bins of the indegree *k_in_*.
(PDF)
**Figure S20**
**Mean number of the four closed triples for nodes with the same indegree in the Flickr social network and the model.** Results for the mean number of closed triples corresponding to (a) *FB*, (b) *FF_a_*, (c) *FF_b_*, and (d) *FF_c_* loops for nodes with the same indegree are shown for the Flickr social network (black squares) and simulated network (red circles) based on the model. Analytic treatment (see Eq. (28)) gives a scaling behavior with an exponent 2, as indicated by the dash line. Data points are averages over the logarithmic bins of the indegree *k_in_*.
(PDF)
**Figure S21**
**Mean number of the four closed triples for nodes with the same indegree in the YouTube social network and the model.** Results for the mean number of closed triples corresponding to (a) *FB*, (b) *FF_a_*, (c) *FF_b_*, and (d) *FF_c_* loops for nodes with the same indegree are shown for the YouTube social network (black squares) and simulated network (red circles) based on the model. Analytic treatment (see Eq. (28)) gives a scaling behavior with an exponent 2, as indicated by the dash line. Data points are averages over the logarithmic bins of the indegree *k_in_*.
(PDF)
**Figure S22**
**Mean number of the four closed triples for nodes with the same outdegree in the** **Slashdot social network and the model.** Results for the mean number of closed triples corresponding to (a) *FB*, (b) *FF_a_*, (c) *FF_b_*, and (d) *FF_c_* loops for nodes with the same outdegree are shown for the Slashdot social network (black squares) and simulated network (red circles) based on the model. Analytic treatment (see Eq. (28)) gives a scaling behavior with an exponent 2, as indicated by the dash line. Data points are averages over the logarithmic bins of the outdegree *k_out_*.
(PDF)
**Figure S23**
**Mean number of the four closed triples for nodes with the same outdegree in the Flickr social network and the model.** Results for the mean number of closed triples corresponding to (a) *FB*, (b) *FF_a_*, (c) *FF_b_*, and (d) *FF_c_* loops for nodes with the same outdegree are shown for the Flickr social network (black squares) and simulated network (red circles) based on the model. Analytic treatment (see Eq. (28)) gives a scaling behavior with an exponent 2, as indicated by the dash line. Data points are averages over the logarithmic bins of the outdegree *k_out_*.
(PDF)
**Figure S24**
**Mean number of the four closed triples for nodes with the same outdegree in the YouTube social network and the model.** Results for the mean number of closed triples corresponding to (a) *FB*, (b) *FF_a_*, (c) *FF_b_*, and (d) *FF_c_* loops for nodes with the same outdegree are shown for the YouTube social network (black squares) and simulated network (red circles) based on the model. Analytic treatment (see Eq. (28)) gives a scaling behavior with an exponent 2, as indicated by the dash line. Data points are averages over the logarithmic bins of the outdegree *k_out_*.
(PDF)
