# Supplementary material for: Emergence of Scale-Free Close-Knit Friendship Structure in Online Social Networks
Source: PLoS One. 2012 Dec 14;7(12):e50702. doi: 10.1371/journal.pone.0050702 (PMC3522705; doi:10.1371/journal.pone.0050702)
Supplement: Appendix SI — Appendix to the manuscript. (PDF) Table S1 The exponents of various distributions obtained by power-law fits of real online social networks and the simulated network based on the model using the maximum likelihood estimation. xmin is the lower bound of the range for fitting a power-law distribution, is the corresponding exponent and KS is the goodness-of-fit value based on the Kolmogorov-Smirnov statistic. (PDF) Table S2 Pearson correlation coefficient. r(in; in) quantifies the tendency of nodes with a high indegree to be connected to another node with a high indegree. The other quantities carry a similar interpretation. (PDF) Figure S1 Indegree (a) and outdegree (b) distributions of the Slashdot social network (black squares) and simulation results (red circles) based on the model. The dashed lines in both panels have a slope −2.1 as the analytic results in Eqs. (17) and (31) suggested. The simulated network is generated by the model with the parameters N = 82168, m ≈5.14 and p ≈0.67 as determined by the mean degree 〈k〉 and reciprocity of the Slashdot social network. Data points are averages over the logarithmic bins of the indegree kin and outdegree kout, respectively. (PDF) Figure S2 Indegree (a) and outdegree (b) distributions of the Flickr social network (black squares) and simulation results (red circles) based on the model. The dashed lines in both panels have a slope −2.08 as the analytic results in Eqs. (17) and (31) suggested. The simulated network is generated by the model with the parameters N = 100000, m ≈8.07 and p ≈0.39 as determined by the mean degree 〈k〉 and reciprocity of the Flickr social network. Data points are averages over the logarithmic bins of the indegree kin and outdegree kout, respectively. (PDF) Figure S3 Indegree (a) and outdegree (b) distributions of the YouTube social network (black squares) and simulation results (red circles) based on the model. The dashed lines in both panels have a slope −2.3 as the analytic results in Eqs. (17) [file pone.0050702.s001.pdf]

Appendix SI: Emergence of scale-free close-knit friendship structure in online social networks

This Appendix is divided into three sections. In Sec. 1, we give the exponents of power-law fits carried out by the maximum likelihood estimation. In Sec. 2, we present the degree correlation by the Pearson correlation coefficient. In Sec. 3, statistical properties of *Slashdot*, *Flickr* and *YouTube* are presented.

Table S1. The exponents of various distributions obtained by power-law fits of real online social networks and the simulated network based on the model using the maximum likelihood estimation. x_{min} **is the lower bound of the range for fitting a power-law distribution,** γ **is the corresponding exponent and** KS **is the goodness-of-fit value based on the Kolmogorov-Smirnov statistic.**

	Epinions			model		
Distribution	γ	x_{min}	KS	γ	x_{min}	KS
$P(k_{in})$	1.73	2	0.018	1.95	3	0.009
$P(k_{out})$	1.71	2	0.022	1.96	3	0.014
$P(k_r)$	1.69	1	0.01	2.1	4	0.025
$P(n_{FB})$	1.37	2	0.034	1.47	3	0.006
$P(n_{FFa})$	1.39	2	0.04	1.46	5	0.009
$P(n_{FFb})$	1.35	2	0.029	1.46	2	0.008
$P(n_{FFc})$	1.38	3	0.038	1.46	5	0.009
	Slashdot			model		
Distribution	γ	x_{min}	KS	γ	x_{min}	KS
$P(k_{in})$	1.67	2	0.045	1.83	3	0.004
$P(k_{out})$	1.64	2	0.047	1.83	3	0.003
$P(k_r)$	1.68	2	0.047	1.85	2	0.004
$P(n_{FB})$	1.55	6	0.027	1.38	4	0.009
$P(n_{FFa})$	1.53	6	0.029	1.38	4	0.015
$P(n_{FFb})$	1.54	6	0.03	1.38	4	0.008
$P(n_{FFc})$	1.56	6	0.034	1.38	4	0.017
	Flickr			model		
Distribution	γ	x_{min}	KS	γ	x_{min}	KS
$P(k_{in})$	1.71	2	0.015	1.8	4	0.004
$P(k_{out})$	1.72	5	0.03	1.8	4	0.005
$P(k_r)$	1.78	5	0.025	1.86	1	0.006
$P(n_{FB})$	1.38	2	0.029	1.36	4	0.007
$P(n_{FFa})$	1.38	5	0.029	1.35	4	0.01
$P(n_{FFb})$	1.37	2	0.029	1.36	4	0.007
$P(n_{FFc})$	1.39	4	0.033	1.36	5	0.009
	YouTube			model		
Distribution	γ	x_{min}	KS	γ	x_{min}	KS
$P(k_{in})$	2.05	3	0.015	2.12	3	0.013
$P(k_{out})$	2.08	6	0.018	2.12	3	0.015
$P(k_r)$	2.06	6	0.019	2.2	3	0.006
$P(n_{FB})$	1.62	4	0.031	1.57	2	0.01
$P(n_{FFa})$	1.62	4	0.044	1.57	4	0.01
$P(n_{FFb})$	1.62	4	0.029	1.57	4	0.005
$P(n_{FFc})$	1.64	4	0.042	1.57	4	0.01

2. Degree correlation measured by Pearson correlation coefficient

Table S2. Pearson correlation coefficient. $r(in, in)$ **quantifies the tendency of nodes with a high indegree to be connected to another node with a high indegree. The other quantities carry a similar interpretation.**

Datasets	$r(in, in)$	$r(in, out)$	$r(out, out)$	$r(out, in)$
Epinions	-0.009	0.073	-0.016	-0.053
Slashdot	-0.068	-0.059	-0.064	-0.071
Flickr	0.06	0.055	-0.0025	-0.001
YouTube	-0.03	-0.032	-0.036	-0.035

3. Statistical properties of *Slashdot*, *Flickr* and *YouTube* social networks

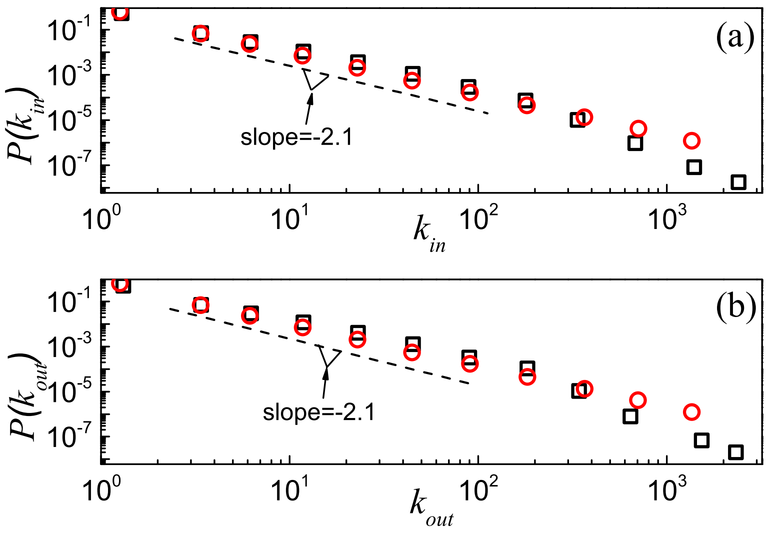

Figure S1. Indegree (a) and outdegree (b) distributions of the Slashdot social network (black squares) and simulation results (red circles) based on the model. The dashed lines in both panels have a slope -2.1 as the analytic results in Eqs. (17) and (31) suggested. The simulated network is generated by the model with the parameters $N = 82168$, $m \approx 5.14$ and $p \approx 0.67$ as determined by the mean degree $\langle k \rangle$ and reciprocity of the Slashdot social network. Data points are averages over the logarithmic bins of the indegree k_{in} and outdegree k_{out}, respectively.

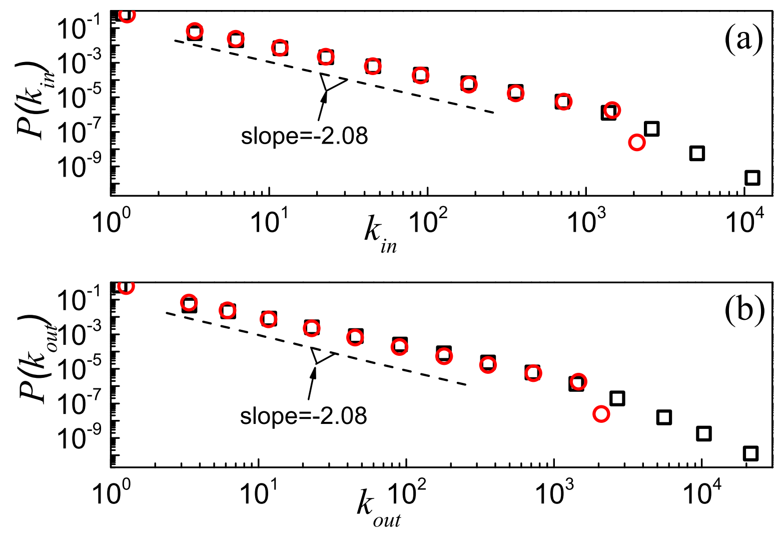

Figure S2. Indegree (a) and outdegree (b) distributions of the Flickr social network (black squares) and simulation results (red circles) based on the model. The dashed lines in both panels have a slope -2.08 as the analytic results in Eqs. (17) and (31) suggested. The simulated network is generated by the model with the parameters $N = 100000$, $m \approx 8.07$ and $p \approx 0.39$ as determined by the mean degree $\langle k \rangle$ and reciprocity of the Flickr social network. Data points are averages over the logarithmic bins of the indegree k_{in} and outdegree k_{out}, respectively.

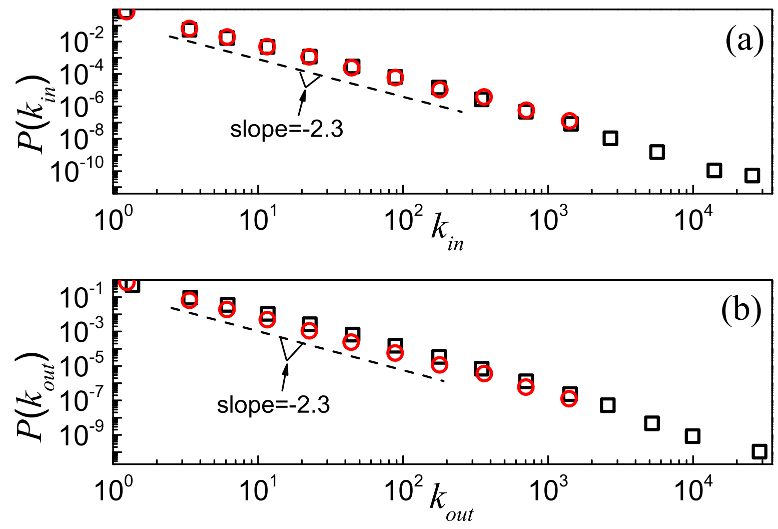

Figure S3. Indegree (a) and outdegree (b) distributions of the YouTube social network (black squares) and simulation results (red circles) based on the model. The dashed lines in both panels have a slope -2.3 as the analytic results in Eqs. (17) and (31) suggested. The simulated network is generated by the model with the parameters $N = 100000$, $m \approx 4.34$ and $p \approx 0.08$ as determined by the mean degree $\langle k \rangle$ and reciprocity of the YouTube social network. Data points are averages over the logarithmic bins of the indegree k_{in} and outdegree k_{out}, respectively.

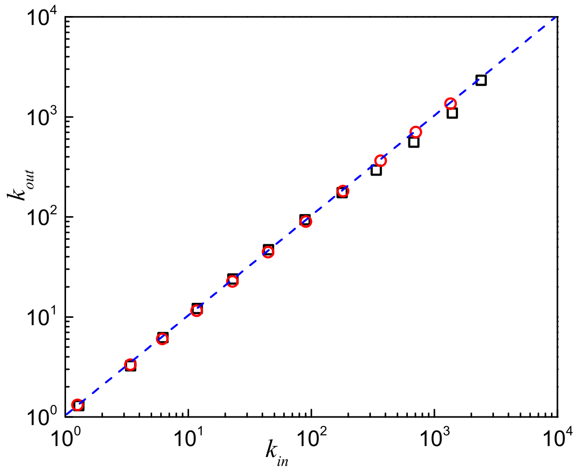

Figure S4. Relationship between the indegree and the outdegree of nodes in the Slashdot social network and the model. Results of the Slashdot social network (black squares) and simulation results (red circles) based on the model are shown. The blue dash line represents the relation function $k_{in} = k_{out}$. Data points are averages over the logarithmic bins of the indegree k_{in}.

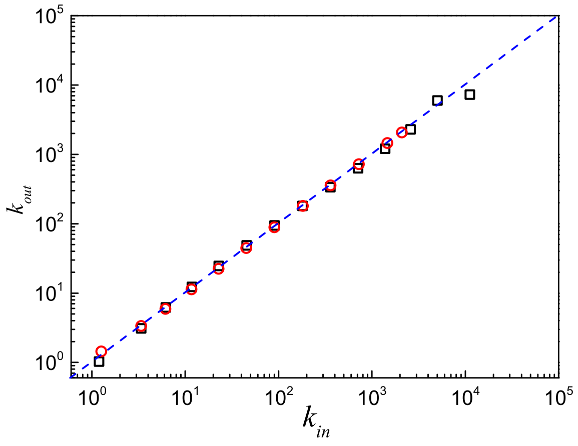

Figure S5. Relationship between the indegree and the outdegree of nodes in the Flickr social network and the model. Results of the Flickr social network (black squares) and simulation results (red circles) based on the model are shown. The blue dash line represents the relation function $k_{in} = k_{out}$. Data points are averages over the logarithmic bins of the indegree k_{in}.

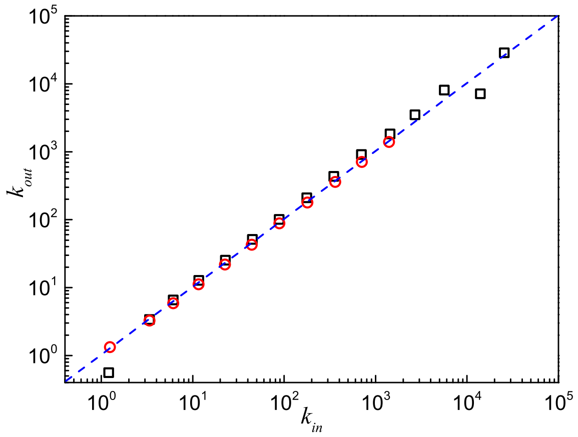

Figure S6. Relationship between the indegree and the outdegree of nodes in the YouTube social network and the model. Results of the YouTube social network (black squares) and simulation results (red circles) based on the model are shown. The blue dash line represents the relation function $k_{in} = k_{out}$. Data points are averages over the logarithmic bins of the indegree k_{in}.

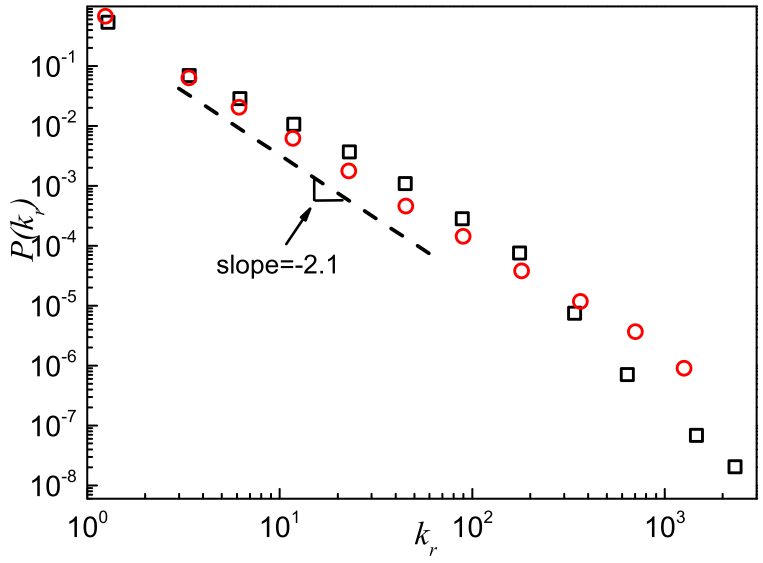

Figure S7. Reciprocal degree distributions of the Slashdot social network and the model. Results of the Slashdot social network (black squares) and simulation results (red circles) based on the model are shown. Analytic treatment (see Eqs. (17) and (31)) suggests a scaling behavior with an exponent -2.1, as shown by the dash line. Data points are averages over the logarithmic bins of the reciprocal degree k_r.

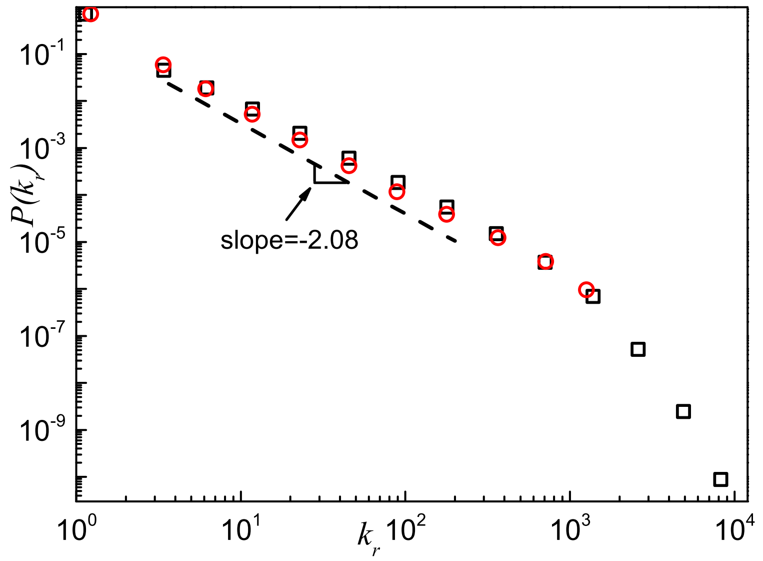

Figure S8. Reciprocal degree distributions of the Flickr social network and the model. Results of the Flickr social network (black squares) and simulation results (red circles) based on the model are shown. Analytic treatment (see Eqs. (17) and (31)) suggests a scaling behavior with an exponent -2.08, as shown by the dash line. Data points are averages over the logarithmic bins of the reciprocal degree k_r.

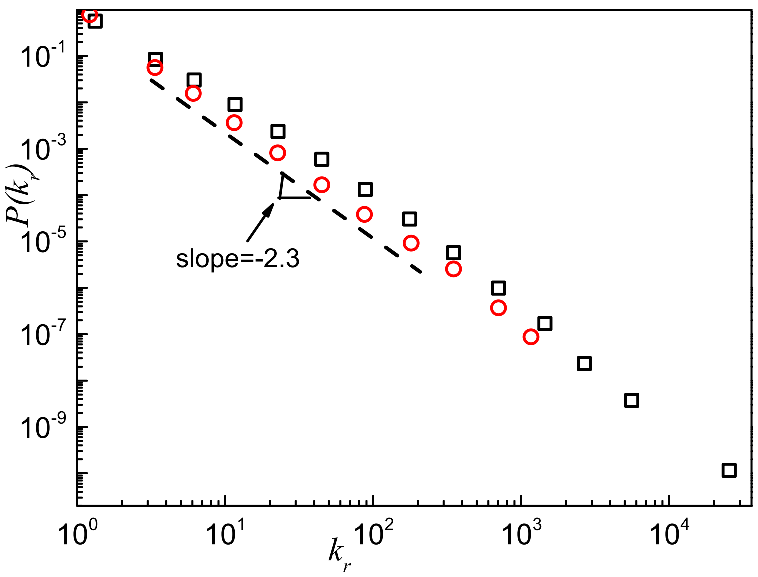

Figure S9. Reciprocal degree distributions of the YouTube social network and the model. Results of the YouTube social network (black squares) and simulation results (red circles) based on the model are shown. Analytic treatment (see Eqs. (17) and (31)) suggests a scaling behavior with an exponent -2.3, as shown by the dash line. Data points are averages over the logarithmic bins of the reciprocal degree k_r.

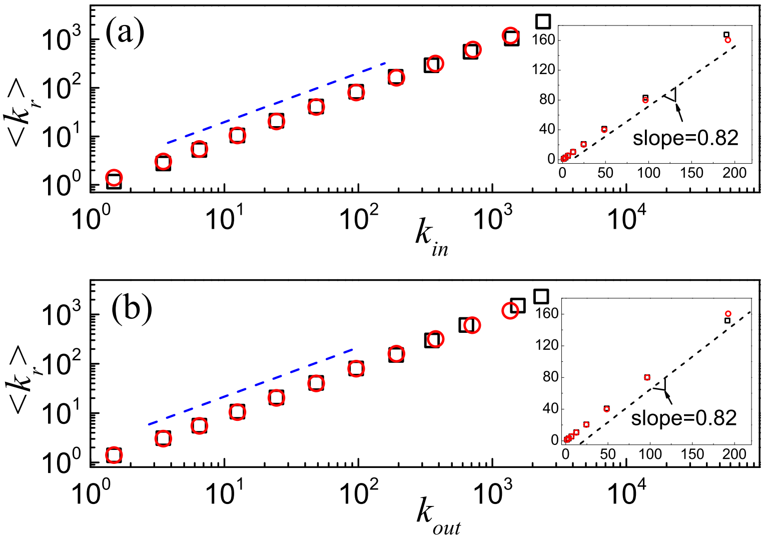

Figure S10. Mean reciprocal degree of nodes with (a) the same indegree and (b) the same outdegree in the Slashdot social network and in the model. Results of the Slashdot social network (black squares) and simulation results (red circles) based on the model are shown in a log-log scale in the main panels. Analytic treatment suggests that $\langle k_r \rangle$ is linearly dependent on k_{in} and k_{out}, and the blue dash lines of slope 1 show its dependence. The inset in each panel shows the results in a linear scale and the dash line has a slope of 0.82, as given by Eqs. (20) and (31). Data points are averages over the logarithmic bins of the indegree k_{in} and outdegree k_{out}, respectively.

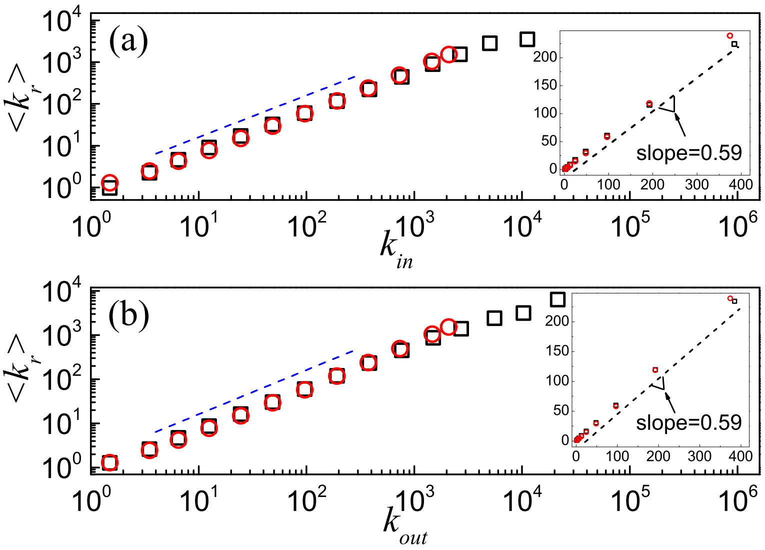

Figure S11. Mean reciprocal degree of nodes with (a) the same indegree and (b) the same outdegree in the Flickr social network and in the model. Results of the Flickr social network (black squares) and simulation results (red circles) based on the model are shown in a log-log scale in the main panels. Analytic treatment suggests that $\langle k_r \rangle$ is linearly dependent on k_{in} and k_{out}, and the blue dash lines of slope 1 show its dependence. The inset in each panel shows the results in a linear scale and the dash line has a slope of 0.59, as given by Eqs. (20) and (31). Data points are averages over the logarithmic bins of the indegree k_{in} and outdegree k_{out}, respectively.

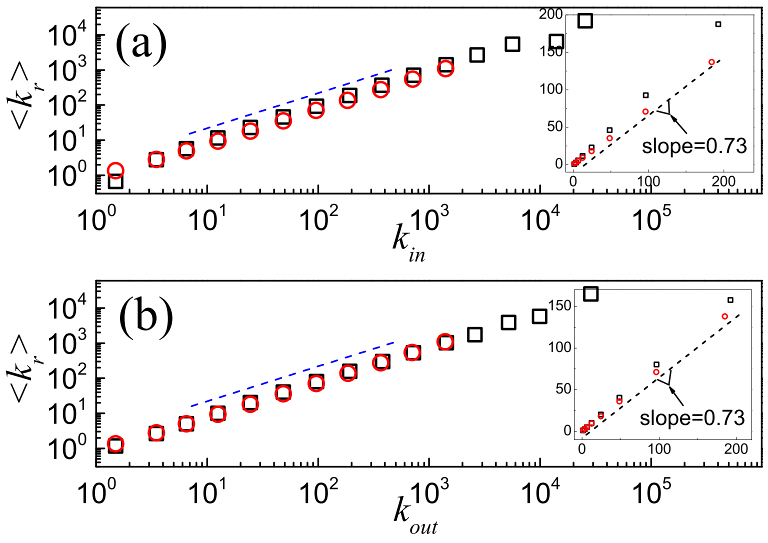

Figure S12. Mean reciprocal degree of nodes with (a) the same indegree and (b) the same outdegree in the YouTube social network and in the model. Results of the YouTube social network (black squares) and simulation results (red circles) based on the model are shown in a log-log scale in the main panels. Analytic treatment suggests that $\langle k_r \rangle$ is linearly dependent on k_{in} and k_{out}, and the blue dash lines of slope 1 show its dependence. The inset in each panel shows the results in a linear scale and the dash line has a slope of 0.73, as given by Eqs. (20) and (31). Data points are averages over the logarithmic bins of the indegree k_{in} and outdegree k_{out}, respectively.

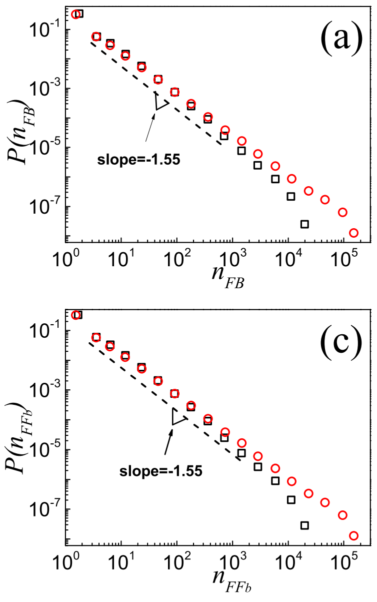

Figure S13. Distributions of four basic closed triples in the slashdot social network and the model. Distributions of closed triples corresponding to (a) FB, (b) FF_a, (c) FF_b, and (d) FF_c loops in the Slashdot social network (black squares) and in the simulated network based on the model (red circles). Analytic treatment (see Eqs. (30) and (31)) suggests a scaling behavior with an exponent -1.55, as shown by the dash lines. Data points are averages over the logarithmic bins of the n_{FB}, n_{FFa}, n_{FFb} and n_{FFc}, respectively.

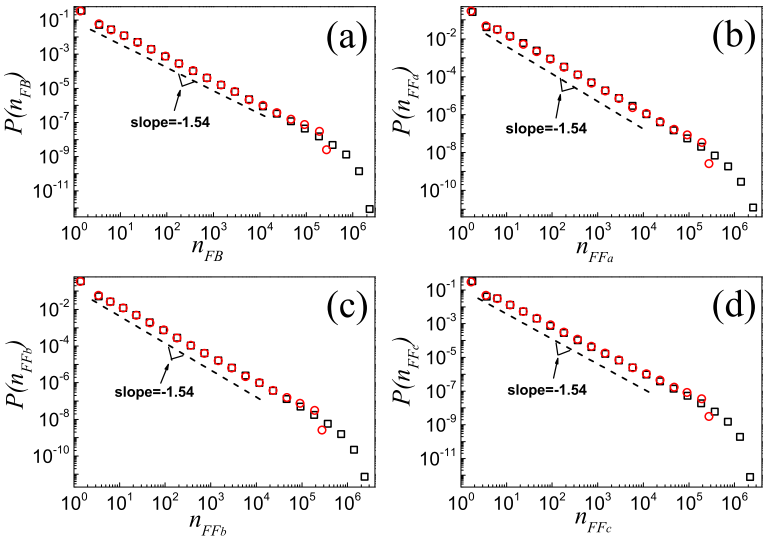

Figure S14. Distributions of four basic closed triples in the Flickr social network and the model. Distributions of closed triples corresponding to (a) FB, (b) FF_a, (c) FF_b, and (d) FF_c loops in the Flickr social network (black squares) and in the simulated network based on the model (red circles). Analytic treatment (see Eqs. (30) and (31)) suggests a scaling behavior with an exponent -1.54, as shown by the dash lines. Data points are averages over the logarithmic bins of the n_{FB}, n_{FFa}, n_{FFb} and n_{FFc}, respectively.

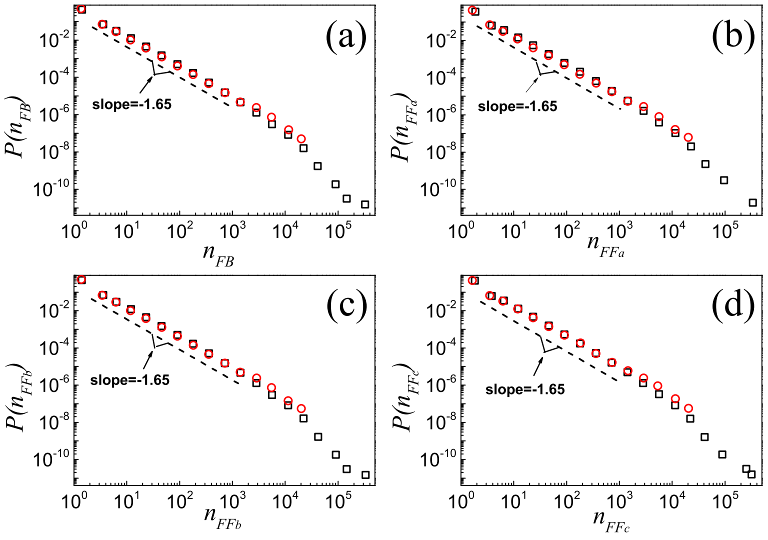

Figure S15. Distributions of four basic closed triples in the YouTube social network and the model. Distributions of closed triples corresponding to (a) FB, (b) FF_a, (c) FF_b, and (d) FF_c loops in the YouTube social network (black squares) and in the simulated network based on the model (red circles). Analytic treatment (see Eqs. (30) and (31)) suggests a scaling behavior with an exponent -1.65, as shown by the dash lines. Data points are averages over the logarithmic bins of the n_{FB}, n_{FFa}, n_{FFb} and n_{FFc}, respectively.

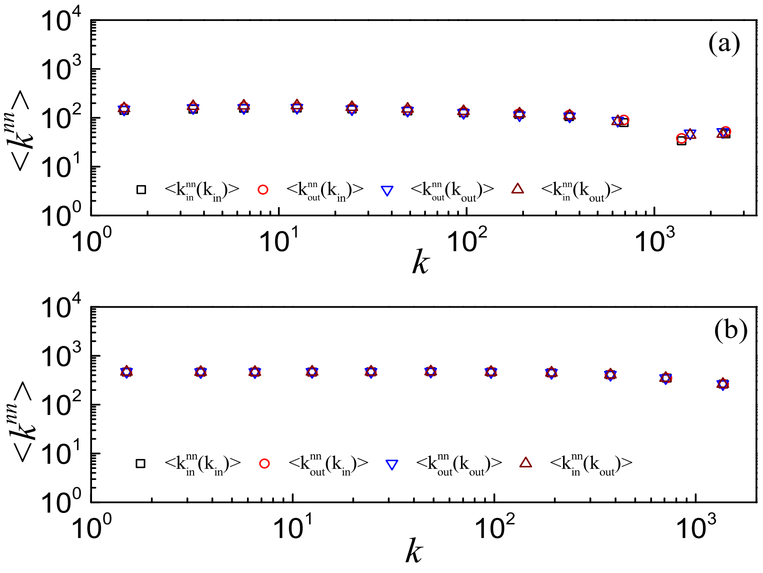

Figure S16. Degree correlations in the Slashdot social network and the model. Results of degree correlations as measured by four quantities corresponding to the average nearest neighbor degree $< k_{in}^{nn}(k_{in}) >$ (squares), $< k_{out}^{nn}(k_{in}) >$ (circles), $< k_{out}^{nn}(k_{out}) >$ (triangles), and $< k_{in}^{nn}(k_{out}) >$ (inverted triangles) for (a) Slashdot social network and (b) simulated network based on the model. Data points are averages over the logarithmic bins of the indegree k_{in} or outdegree k_{out}.

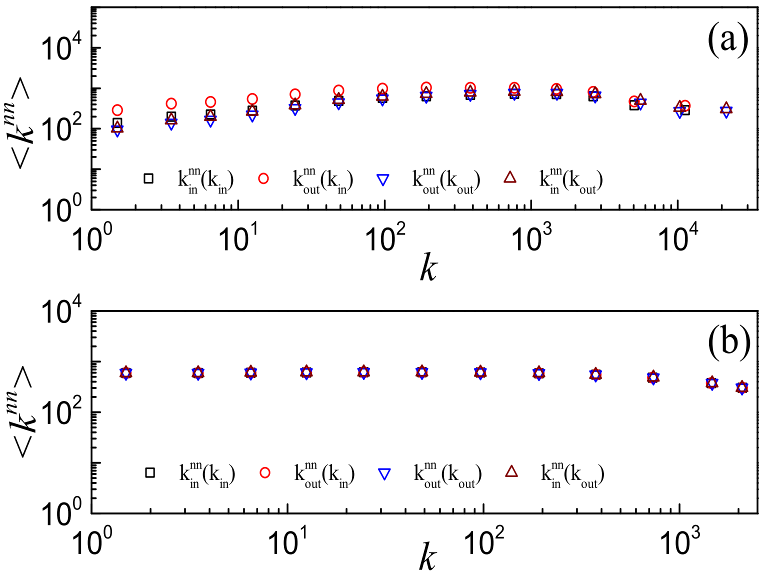

Figure S17. Degree correlations in the Flickr social network and the model. Results of degree correlations as measured by four quantities corresponding to the average nearest neighbor degree $< k_{in}^{nn}(k_{in}) >$ (squares), $< k_{out}^{nn}(k_{in}) >$ (circles), $< k_{out}^{nn}(k_{out}) >$ (triangles), and $< k_{in}^{nn}(k_{out}) >$ (inverted triangles) for (a) Flickr social network and (b) simulated network based on the model. Data points are averages over the logarithmic bins of the indegree k_{in} or outdegree k_{out}.

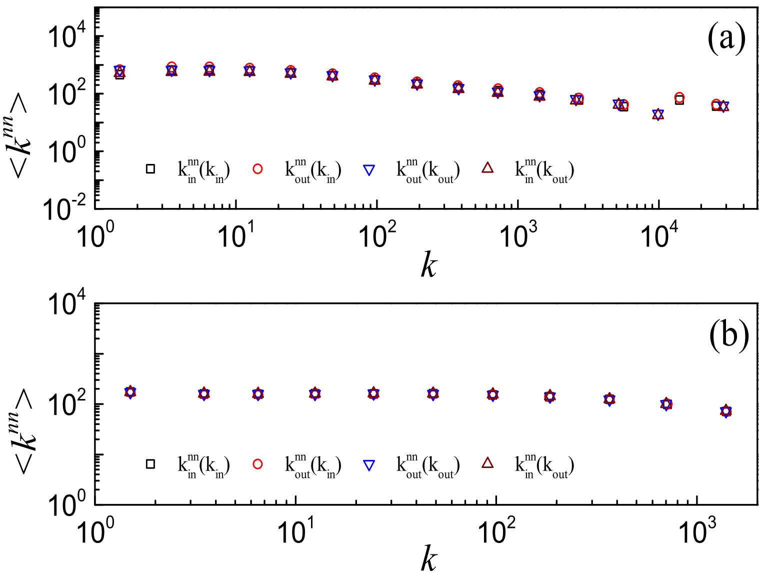

Figure S18. Degree correlations in the YouTube social netowrk and the model. Results of degree correlations as measured by four quantities corresponding to the average nearest neighbor degree $< k_{in}^{nn}(k_{in}) >$ (squares), $< k_{out}^{nn}(k_{in}) >$ (circles), $< k_{out}^{nn}(k_{out}) >$ (triangles), and $< k_{in}^{nn}(k_{out}) >$ (inverted triangles) for (a) YouTube social network and (b) simulated network based on the model. Data points are averages over the logarithmic bins of the indegree k_{in} or outdegree k_{out}.

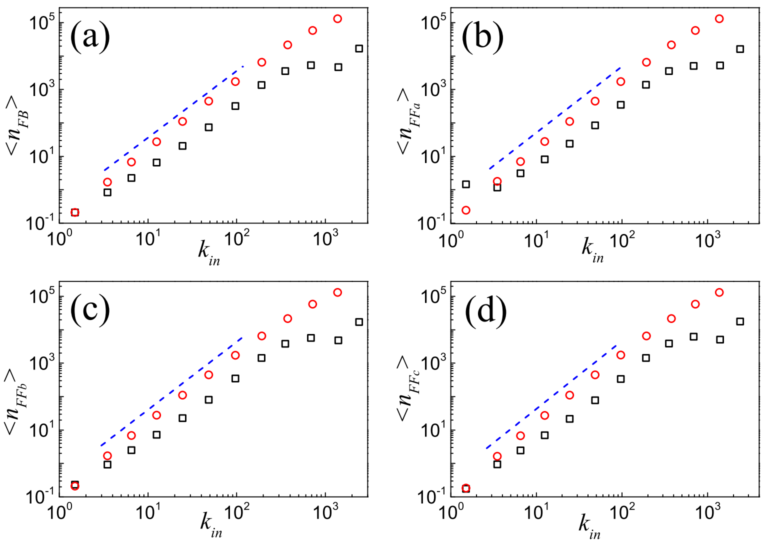

Figure S19. Mean number of the four closed triples for nodes with the same indegree in the Slashdot social network and the model. Results for the mean number of closed triples corresponding to (a) FB, (b) FF_a, (c) FF_b, and (d) FF_c loops for nodes with the same indegree are shown for the Slashdot social network (black squares) and simulated network (red circles) based on the model. Analytic treatment (see Eq. (28)) gives a scaling behavior with an exponent 2, as indicated by the dash line. Data points are averages over the logarithmic bins of the indegree k_{in}.

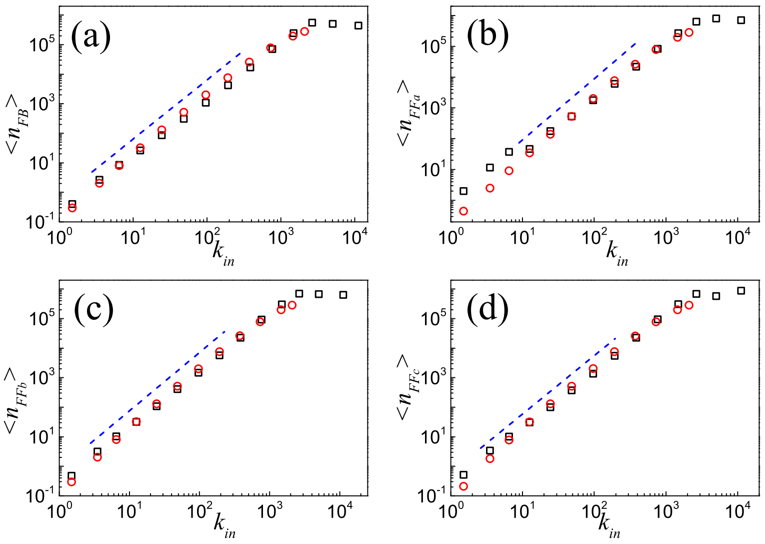

Figure S20. Mean number of the four closed triples for nodes with the same indegree in the Flickr social network and the model. Results for the mean number of closed triples corresponding to (a) FB, (b) FF_a, (c) FF_b, and (d) FF_c loops for nodes with the same indegree are shown for the Flickr social network (black squares) and simulated network (red circles) based on the model. Analytic treatment (see Eq. (28)) gives a scaling behavior with an exponent 2, as indicated by the dash line. Data points are averages over the logarithmic bins of the indegree k_{in}.

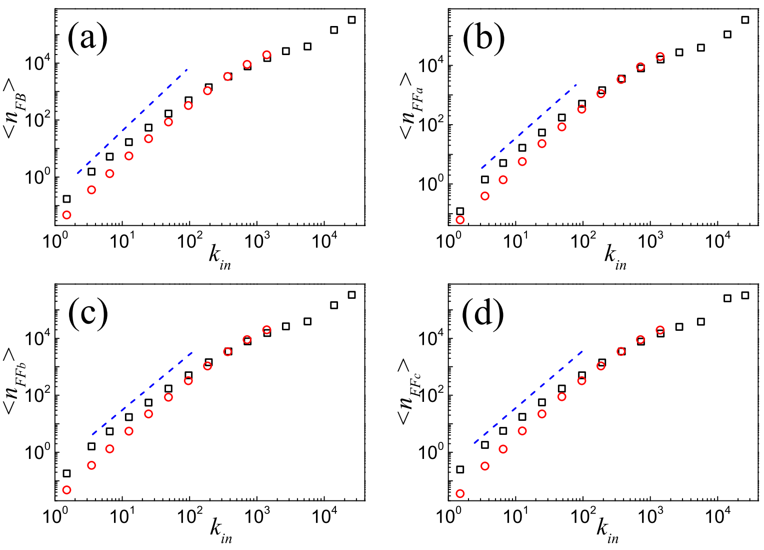

Figure S21. Mean number of the four closed triples for nodes with the same indegree in the YouTube social network and the model. Results for the mean number of closed triples corresponding to (a) FB, (b) FF_a, (c) FF_b, and (d) FF_c loops for nodes with the same indegree are shown for the YouTube social network (black squares) and simulated network (red circles) based on the model. Analytic treatment (see Eq. (28)) gives a scaling behavior with an exponent 2, as indicated by the dash line. Data points are averages over the logarithmic bins of the indegree k_{in}.

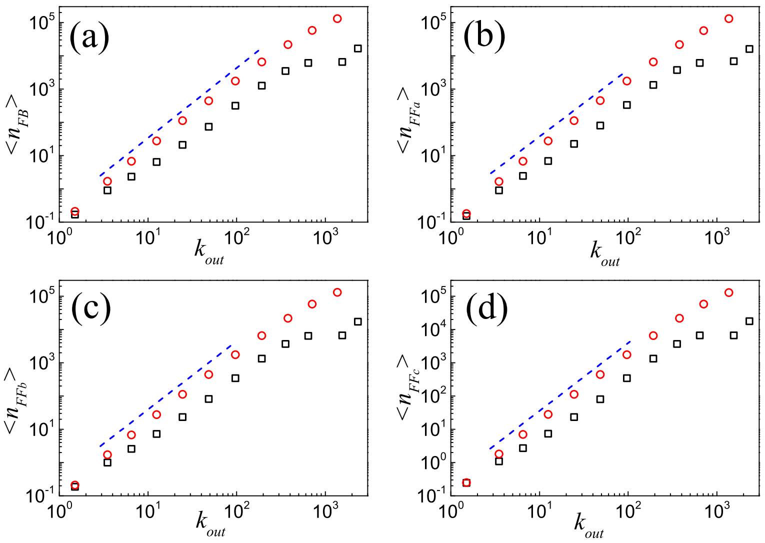

Figure S22. Mean number of the four closed triples for nodes with the same outdegree in the Slashdot social network and the model. Results for the mean number of closed triples corresponding to (a) FB, (b) FF_a, (c) FF_b, and (d) FF_c loops for nodes with the same outdegree are shown for the Slashdot social network (black squares) and simulated network (red circles) based on the model. Analytic treatment (see Eq. (28)) gives a scaling behavior with an exponent 2, as indicated by the dash line. Data points are averages over the logarithmic bins of the outdegree k_{out}.

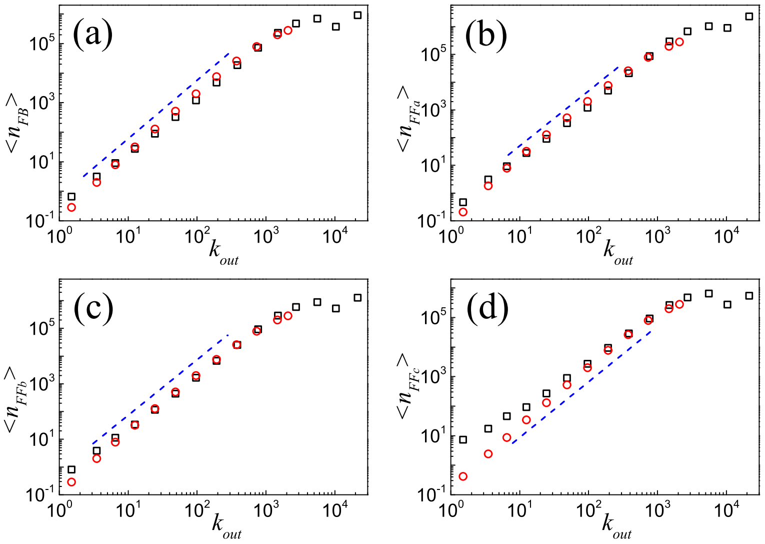

Figure S23. Mean number of the four closed triples for nodes with the same outdegree in the Flickr social network and the model. Results for the mean number of closed triples corresponding to (a) FB, (b) FF_a, (c) FF_b, and (d) FF_c loops for nodes with the same outdegree are shown for the Flickr social network (black squares) and simulated network (red circles) based on the model. Analytic treatment (see Eq. (28)) gives a scaling behavior with an exponent 2, as indicated by the dash line. Data points are averages over the logarithmic bins of the outdegree k_{out}.

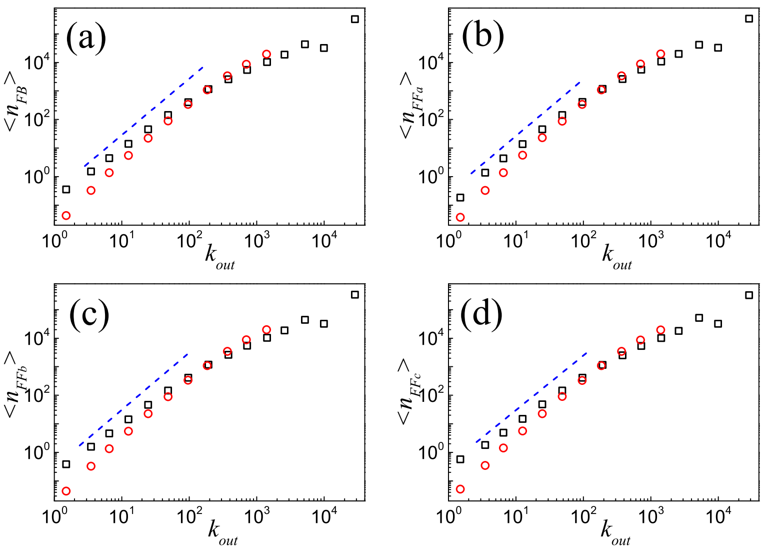

Figure S24. Mean number of the four closed triples for nodes with the same outdegree in the YouTube social network and the model. Results for the mean number of closed triples corresponding to (a) FB, (b) FF_a, (c) FF_b, and (d) FF_c loops for nodes with the same outdegree are shown for the YouTube social network (black squares) and simulated network (red circles) based on the model. Analytic treatment (see Eq. (28)) gives a scaling behavior with an exponent 2, as indicated by the dash line. Data points are averages over the logarithmic bins of the outdegree k_{out}.
